# Unique Transcriptional Signatures Correlate with Behavioral and Psychological Symptom Domains in Alzheimer’s Disease

**DOI:** 10.21203/rs.3.rs-2444391/v1

**Published:** 2023-01-11

**Authors:** Daniel W. Fisher, Jeffrey T. Dunn, Rachel Keszycki, Guadalupe Rodriguez, David A. Bennett, Robert S. Wilson, Hongxin Dong

**Affiliations:** 1 Department of Psychiatry and Behavioral Sciences, Northwestern University Feinberg School of Medicine; 2 Department of Psychiatry and Behavioral Sciences, University of Washington School of Medicine; 3 Mesulam Center for Cognitive Neurology and Alzheimer’s Disease, Northwestern University Feinberg School of Medicine; 4 Rush Alzheimer’s Disease Center, Rush University Medical Center

**Keywords:** Alzheimer’s disease, Behavioral and psychological symptoms of dementia, Neuropsychiatric symptoms, Transcriptome, RNA-seq, Anterior cingulate cortex, Apathy, Depression, Anxiety, Impulsivity, Disinhibition, Aggression, Psychosis

## Abstract

Despite the significant burden, cost, and worse prognosis of Alzheimer’s disease (AD) with behavioral and psychological symptoms of dementia (BPSD), little is known about the molecular causes of these symptoms. Using antemortem assessments of BPSD in AD, we demonstrate that individual BPSD can be grouped into 4 domain factors in our sample: affective, apathy, agitation, and psychosis. Then, we performed a transcriptome-wide analysis for each domain utilizing bulk RNA-seq of post-mortem anterior cingulate cortex (ACC) tissue. Though all 4 domains are associated with a predominantly downregulated pattern of hundreds of differentially expressed genes (DEGs), most DEGs are unique to each domain, with only 22 DEGs being common to all BPSD domains, including *TIMP1*. Weighted gene co-expression network analysis (WGCNA) yielded multiple transcriptional modules that were shared between BPSD domains or unique to each domain, and NetDecoder was used to analyze context-dependent information flow through the biological network. For the agitation domain, we found that all DEGs and a highly correlated transcriptional module were functionally enriched for ECM-related genes including *TIMP1, TAGLN*, and *FLNA*. Another unique transcriptional module also associated with the agitation domain was enriched with genes involved in post-synaptic signaling, including *DRD1, PDE1B, CAMK4*, and *GABRA4*. By comparing context-dependent changes in DEGs between cases and control networks, *ESR1* and *PARK2* were implicated as two high impact genes associated with agitation that mediated significant information flow through the biological network. Overall, our work establishes unique targets for future study of the biological mechanisms of BPSD and resultant drug development.

## INTRODUCTION

Although dementia is defined largely by memory and cognitive decline which results in the loss of the ability to function independently, behavioral and psychological symptoms in dementia (BPSD) play a large role in a patient’s overall functional level [1]. Significant BPSD are more the rule than the exception, as >90% of people with dementia develop BPSD during the disease course[2, 3]. BPSD encompass a wide array of symptoms and include aggression, agitation, hyperactivity, compulsions, disinhibition, anxiety, depression and dysphoria, euphoria delusions, and hallucinations. In addition, there is evidence that mild behavioral impairment (MBI) is the first sign of dementia in some people, analogous to the more widely recognized mild cognitive impairment (MCI)[4, 5]. In agreement with this, neuropsychiatric symptoms can precede a dementia diagnosis[4, 6], with some estimates suggesting that over half demonstrate neuropsychiatric symptoms before a diagnosis of a cognitive disorder, including MCI[7]. Neuropsychiatric symptoms are also associated with faster cognitive decline in cognitively unimpaired individuals[8-10] and those with MCI[11], quicker development of dementia in people with MCI[12-14], and faster dementia progression[15]. In addition, BPSD result in poorer quality of life for those with dementia[16, 17] as well as significant caregiver distress, often greater than with cognitive deficits[18, 19]. Emergent and difficult to treat, BPSD are often the cause of hospitalization and institutionalization for persons with dementia[20, 21]. Behavioral interventions remain first-line for treating any BPSD, and there is still no FDA approved medications that are indicated to treat any BPSD[22].

BPSD are a heterogeneous group of symptoms, and while each symptom has its own frequency of presentation, studies have suggested that certain symptoms co-occur at greater rates than others[23, 24]. In particular, a systematic review of 62 studies utilizing unbiased clustering of BPSD generally found that affective symptoms (dysphoria, anxiety), apathy, hyperactivity-impulsivity-disinhibition-agitation-aggression (HIDA), and psychosis (delusions, hallucinations) all tend to form independent clusters[24]. This could implicate similar molecular mechanisms underlying each cluster.

Despite the ubiquity and burden of BPSD clinically, very few molecular studies exist to elucidate the molecular mechanisms underlying BPSD[25-27], with the exception of AD with psychosis[28-30]. To begin to identify the molecular mechanisms of BPSD, in this study, we first verified the occurrence of BPSD domains in a sample of older adults based on the use of a structured clinical interview administered within two years of death. Then, we confirmed clustering of BPSD into four predominant domains and then performed bulk RNA-seq analysis on the post-mortem anterior cingulate cortex (ACC) from a subset of those with AD and varying burdens of BPSD. Finally, we performed weighted gene co-expression network analysis (WGCNA) to identify transcriptional clusters associated with each BPSD domain. Finally, we used an algorithm to assess context-dependent information flow differences between the transcriptional networks of cases and controls to determine likely molecular drivers behind each BPSD domain. Our data set to better understand these four BPSD domains on the transcriptional level and yield promising targets for future mechanistic studies and novel therapeutics.

## METHODS

### Subjects

Community-dwelling older adults who later developed dementia and their informants were recruited through the Rush Alzheimer’s Disease Center (RADC) memory clinic. As a part of their research visits, trained research assistants conducted standardized clinical interviews via telephone with informants, including assessment of the frequency and severity of numerous BPSD. Questions pertaining to BPSD were developed by two neuropsychologists from clinical descriptions in the literature, observations of patient behaviors, and caregiver interviews, as described previously[31-34].

This study was approved by an Institutional Review Board of Rush University Medical Center (RUMC). For all subjects, consent for brain autopsy was obtained after death from next of kin and a witness by RUMC staff. Inclusion criteria for subjects in the present study were based on the NINCDS-ADRDA clinical criteria for “probable AD” with diagnosticians blinded to post-mortem findings[35]. Namely, all subjects had a history of cognitive decline, impairment in memory and at least one other cognitive domain, and no other conditions judged to be probably contributing to cognitive impairment (e.g., stroke, Parkinson’s disease). Following autopsy, all subjects underwent neuropathological evaluation by a neuropathologist who was blinded to clinical diagnosis. Subjects received a modified (i.e., dichotomized) NIA-Reagan score based on Braak staging of neurofibrillary tangles and CERAD scoring of neuritic plaques[36, 37]. In total, we identified 192 subjects with probable AD dementia due to primary AD neuropathologic change.

### BPSD domains

To determine the grouping of BPSD into domains, we performed a principal component analysis with oblimin rotation on responses to BPSD questions (Supplementary Table S1) from 192 patients with dementia due to AD. We used a scree plot and Horn’s Parallel Analysis to determine how many components to retain (Supplementary Fig. S1).

### Sample selection for molecular analyses

To best capture relationships between antemortem BPSD and transcriptional changes, we only considered samples from subjects with BPSD data collected within 2 years of death. Of the initial 192 subjects, we identified 100 subjects who met this criterion and had tissue available for sequencing. We created a scoring system to estimate BPSD burden for each of these 100 subjects using an additive composite of individual symptom severity and frequency within each domain. For questions with answer choices of no or yes, a score of 0 or 1 was assigned for each question, respectively. For questions with a frequency component, a low or no frequency received score of 0, a moderate frequency a score of 0.5, and a high frequency a score of 1. The individual question scores were added together to give a final score for each BPSD domain. This yielded four BPSD burden scores for each individual, one for each domain. Within each domain, we used these burden scores to identify patients who were cases (≥70^th^ percentile), controls (≤30^th^ percentile), or neither (31^st^-69^th^ percentile), and used these groupings for subsequent transcriptome analyses. This design permitted an individual subject to be considered as a case for one domain but as a control for another, maximizing the sample sizes of cases and controls for each domain. In choosing a subset of subjects for our final sample, we made sure that each domain contained similar proportions of cases to controls and of males to females so as to minimize bias due to overrepresentation of any particular group. We ultimately chose 60 subjects for biochemical analyses (Table 1).

### RNA Isolation and Sequencing

We confirmed four predominant BPSD clusters and then performed bulk RNA-seq analysis on the post-mortem ACC from a subset of 60 individuals with AD with varying burdens of BPSD. Total RNA was isolated using the QIAGEN RNeasy column-based purification kit (Germantown, MD). The quality of RNA was measured using an Agilent Bioanalyzer, which produces an RNA Integrity Number (RIN) between 1 and 10, with 10 being the highest quality samples showing the least degradation. The RINs of the 60 samples ranged between 5.3-10.0 (88% >7.0), and 1ég of high-quality RNA per sample was used for the total RNA-Seq library preparation. RNA-Seq was conducted at the Northwestern University NUSeq Core Facility. Briefly,_the Illumina TruSeq Stranded Total RNA Library Preparation Kit was used to prepare sequencing libraries. The Kit procedure was performed without modifications. This procedure includes rRNA depletion, remaining RNA purification and fragmentation, cDNA synthesis, 3' end adenylation, Illumina adapter ligation, and library PCR amplification and validation. Illumina HiSeq 4000 Sequencer was used to sequence the libraries with the production of single-end 50 bp reads.

### RNA-seq Differential Expression Analysis

Raw data was pre-processed with TrimGalore, including an initial quality control (QC). Read depth ranged from 50 – 90M. Pseudo-alignment was performed with Kallisto[38] with k-mer 17 due to short read length (50bp). Genes were pseudoaligned to Genome Reference Consortium Human Build 38. Genes with >80% of samples with total counts <5 were removed. Principal variable component analysis (PVCA)[39] was used to identify likely important covariates by identifying factors that explained a significant proportion of variance.

RIN, sex, and RNA-isolation batch were identified as contributing significantly to variation and were included in the final model, while other variables including post-mortem interval, Braak, CERAD, NIA-Reagen scores, and age at death did not. This was also analyzed with Eigen-R2[40], which largely corroborated the PVCA conclusions. Principal component analysis (PCA) was performed and the first two principal components were visualized in scatter plot to identify likely outliers. Clustering analysis within WGCNA was also performed and agreed that one sample was a clear outlier and not used in subsequent analyses, leading to 59 total samples analyzed. Final n per group are as follows: Affective domain, n_control_ = 23, n_case_ = 22; Apathy domain, n_control_ = 23, n_case_ = 25; Agitation domain, n_control_ = 28, n_case_ = 23; Psychosis domain, n_control_ = 24, n_case_ =21. Because overlapping design allowed for individual samples to be analyzed as a case or control depending on their individual domain score, the sum of all comparisons should not add up to the total 59 included samples. We were underpowered to perform differential expression analysis by sex, and therefore performed 4 independent analyses, one for each domain, using the cases and controls identified by our pre-mortem scoring system. DESeq2[41] was used to perform differential expression analysis, and a liberal cutoff value of nominal p < 0.05 and fold-change > 0.2 was used to identify DEGs. Certain DEGs with unusually high variance and fold change were inspected for outliers, and if an isolated datapoint was >3 standard deviations from the mean, the mean was imputed for that point. Visualization of DEG overlap was facilitated with R package VennDiagram.

### Functional Enrichment Analysis

Functional Enrichment Analysis was performed using gProfiler2[42], and is described in more detail in supplementary materials.

### Cellular Decomposition

Prior to cellular decomposition, WGCNA, and NetDecoder analyses, counts were converted to transcripts per million (TPM) and underwent covariate correction and variance stabilizing transformation via limma[43]. BRETIGEA[44] was then performed to estimate the relative abundance of 6 different cell types – Astrocytes, Endothelial Cells, Microglia, Neurons, Oligodendrocytes, and Oligodendrocyte Precursor Cells (OPCs) – using 50 different gene markers per cell type. These results were verified with BisqueMarker[45], which agreed with did not yield significantly different trends in predicted cell type composition.

### WGCNA

Weighted gene co-expression network analysis (WGCNA) was performed to identify modules of gene co-expression[46, 47]. We included only the top 20% most variable genes by overall expression. Details regarding WGCNA optimization and execution are described more fully in supplementary materials. A potential hub gene was defined similar to previous guidance by WGCNA creators as having a module membership (MM) >0.8 and Gene Significance (GS) of >0.2. The notable hub genes in Fig. 3B were identified based on considerations of their high MM, high GS, overall high fold change in expression between cases and controls, and significant presence in the literature as affecting either behaviors in a particular domain or relevance to AD pathogenesis.

### RNA Fluorescent Barcoding

RNA fluorescent barcoding was used to perform multiplex measurement of 37 agitation domain genes of interest (GoIs), the selection of which was informed by WGCNA and differential expression analysis. A custom CodeSet/ProbeSet (NanoString Technologies, Seattle, WA) was designed to measure GoI transcript counts from the RNA samples that remained available following use for RNAseq (N = 47). In addition to GoIs, five reference genes (*IMPDH2*, *LAMTOR1*, *MTFR1L*, *SMIM7*, *TMEM50B*) were selected based on low covariance between cases and controls in our RNA-seq experiment. Eight negative controls and six positive controls (NanoString Technologies) were measured as a component of QC.

Hybridization of reporter and capture probes to the RNA samples was conducted in accordance with the manufacturer’s protocol (NanoString Technologies, MAN-10056-04). Briefly, 50ng of total RNA at a concentration of 10ng/μL was incubated with a Reporter CodeSet-hybridization buffer (NanoString Technologies, item no. 000136) master mix and Capture ProbeSet in a thermocycler at 65°^C^ for 24 hours.

Incubation temperature was then reduced to 4°^C^ until sample processing on the following day. Hybridized samples were brought to a volume of 30μL with RNAse-free water and loaded into an nCounter SPRINT cartridge (NanoString Technologies, item no. 100078), which was run on an nCounter SPRINT profiler. Transcript counts detected by barcode visualization in the nCounter SPRINT profiler were analyzed using nSolver Analysis Software (v4.0). Differences between group means were evaluated using Welch-Satterthwaite t-tests and a threshold of *p* < .05 was implemented for determination of statistical significance. All 47 measured samples were included for analysis, as binding density QC indicated sufficient RNA abundance without lane oversaturation, no fields of view were lost during imaging, and assessment of positive control linearity yielded r^2^ = 1.0 for each sample.

### NetDecoder

NetDecoder was performed to compare context-dependent changes in information-flow through case and control networks, as previously described[48]. A fuller description of NetDecoder is provided in supplementary materials. Briefly, we defined DEGs for each domain as source genes, and used iRefIndex v14.0 to build our interaction network. We presented the top 20 positive and top 20 negative genes in terms of flow difference or impact score for visualization across the three intermediary gene types. For visualizing changes in overall domain networks or subnetworks relating to the intermediary genes, we used Cytoscape. To simplify visualization, we filtered out edges where there was very little difference in flow, thus highlighting the results with the largest effects on the networks.

## RESULTS

### BPSD segregate into four domains

**Fig. 1:**
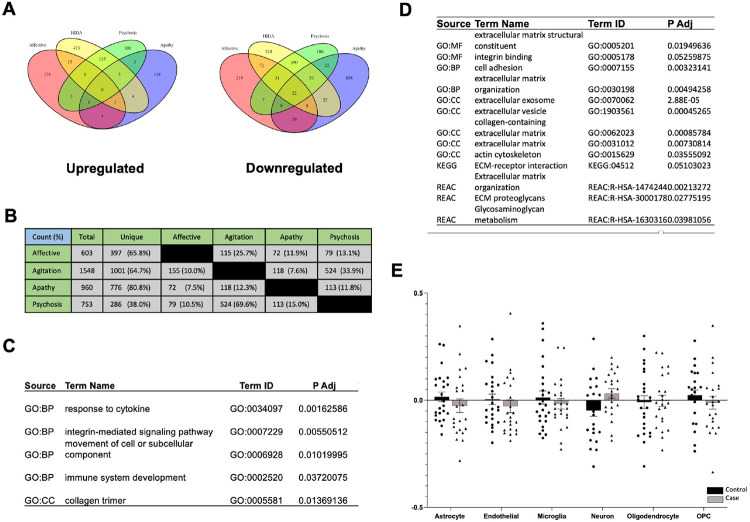
Each BPSD domain is associated with unique differentially expressed genes. **A)** Venn diagrams detailing overlap between DEGs in each domain. DEGs were defined by a fold change >0.20 and a nominal p-value < 0.05. **B)** Table depicting numbers of DEGs that are either shared between two domains or uniquely expressed. Percentages represent how many DEGs are in each category compared to the total number of DEGs. **C)** Functional gene enrichment analysis (FGEA) of the 22 DEGs that were commonly downregulated in all four BPSD domains. Most salient pathways were chosen for this table. **D)** For the agitation domain, FGEA of all DEGs associated with this domain. Most salient pathways were chosen for this table. **E)** Relative cell abundance estimated with BRETIGIA. After correcting for multiple comparisons, there were no differences in cell type between cases and controls for the Agitation Domain (p < 0.05).

Though BPSD are heterogeneous, previous reports have indicated that common symptoms often co-occur at high rates and can be grouped into domains[23, 24]. However, while groupings for BPSD are generally consistent across studies, there are slight variations that could be related to the specific cohort studied (i.e. community, nursing home, assisted living facility, etc.) or stochasticity. We performed clustering analysis of BPSD based on data from a clinical cohort where the frequency and severity of individual BPSD within 2 years from the patients’ deaths was recorded. We determined grouping of BPSD into the following four domains: affective (depression and anxiety), psychosis (predominantly hallucinations), agitation (including aggression), and apathy (Fig. 1). The proportion of variance explained by each factor were comparatively similar. Delusions tended not to be explained by any single loading factor, so these were not included in the psychosis domain, although these are often grouped together clinically and in prior factor analyses[24].

### Unique molecular signatures associate with each BPSD domain

After demonstrating BPSD could be split into 4 domains, we created a scoring system to estimate BPSD burden using a composite of individual symptom severity and frequency within each domain and grouped individual as cases or controls for each domain. Though BPSD are likely to result from dysfunction of multiple brain regions, the ACC has repeatedly been implicated as being involved in all four BPSD domains[49-51]. Therefore, we performed bulk RNA-seq on a subset of individuals from our cohort that maximized the numbers of individuals who could be considered a case or control (Supplementary Table 1).

Using a liberal cut-off for significance of nominal p < 0.05 and fold change > 0.20, we identified hundreds of DEGs for each BPSD domain with a pattern towards downregulated BPSD for all four domains. Specifically, we found 207 upregulated and 396 downregulated (66% of total) DEGs for the affective domain; 135 upregulated and 788 downregulated (86%) DEGs for the apathy domain; 619 upregulated and 931 downregulated (60%) DEGs for the agitation domain; and 227 upregulated and 519 (70%) downregulated for the psychosis domain (Fig. 1A).

Importantly, despite an experimental design using the same subjects as cases and controls on a domain-by-domain basis, most of the DEGs were transcriptionally unique for each domain (Fig. 1B): 65.8% unique for the affective domain, 80.8% unique for the apathy domain, and 64.7% unique for the agitation domain. The exception was the psychosis domain, where only 38.0% of DEGs were unique due to high overlap with DEGs from the agitation domain.

Transcriptional signatures can be used to estimate relative cell abundance of the origin tissue. Therefore, we used BRETIGEA to estimate the cell abundance of 6 major cell types – astrocytes, endothelial cells, microglia, neurons, oligodendrocytes, and oligodendrocyte precursor cells. Across all four domains, only a decrease in microglia was detected for cases compared to controls in the apathy domain, while all other cell type compositions were comparable (p < 0.05). The results were similar when a different deconvolution algorithm, BisqueMarker, was used (data not shown).

Interestingly, there were only 22 DEGs that were shared by all four BPSD domains (<3.6% of total DEGs in any domain) and all were downregulated. Functional enrichment analysis yielded pathways related to response to cytokines, integrin-mediated signaling, and collagen trimers (Fig. 1C), with *TIMP1* being a notable DEG (M_Fold Change, agitation_.= 0.644, p = 3.0 x 10^−5^; M_FC, affective_ = 0.748, p = 0.012; M_FC, apathy_.= 0.644, p = 1.1 x 10^−5^; M_FC, psychosis_.= 0.642, p = 1.1 x 10^−5^).

Functional enrichment analysis of the DEGs were performed for each BPSD domain. Here, we focus on the results of the agitation domain, as this domain is often most challenging for families and clinicians due to high risk of safety issues including aggression, increased risk-taking and impulsivity, etc. (see Supplementary Materials for results and discussion of the other 3 BPSD domains). We found an enrichment for the extracellular matrix (ECM) including actin, collagen, glycosaminoglycans, extracellular vesicles, and cellular adhesion (Fig. 1D). Transcriptomic changes detected in agitation cases did not coincide with differences in the abundance of any individual cell type (Fig. 1E).

**Fig. 2:**
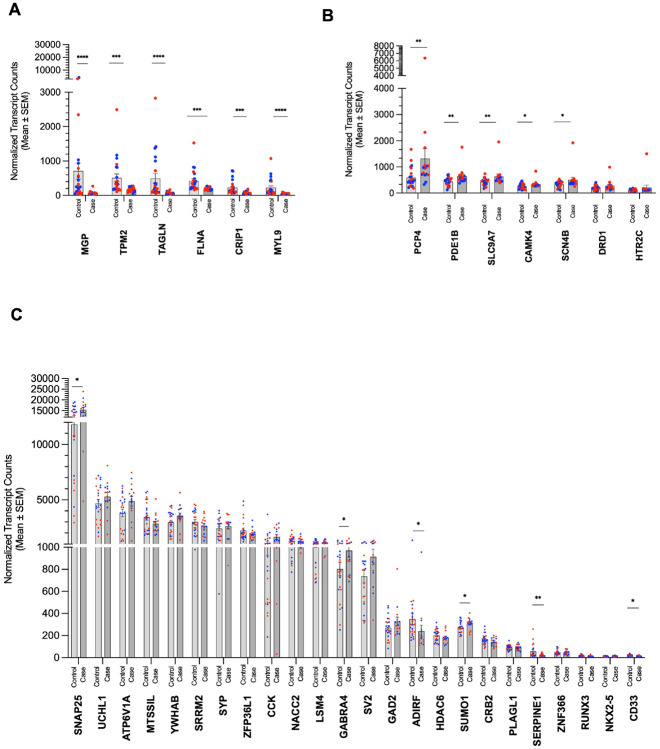
Validation of key DEGs for the agitation domain. The differential expression of **A)** Six DEGs that are also potential hub genes in the ECM (Darkturquoise) module and **B)** Five DEGs that are potential hub genes in the post-synapse (Darkgrey) module were confirmed with RNA fluorescent barcoding after expression was normalized to five housekeeping genes. **C)** Non-WGCNA genes and modules with a single confirmed potential hub gene (i.e., *ADIRF* from the nucleosome (Lightyellow) module and *CD33* from the transcription factor (Magenta) module) are represented. Blue circle = male, red circle = female; **** = p < 0.0001, *** = p < 0.001, ** = p <0 .01, * = p < 0.05.

To confirm some of the results for our agitation domain, we quantitated transcripts using Nanostring. Though our statistical power was more limited than our initial RNAseq, we were able to confirm 17/37 genes to be significantly different between cases and controls for the agitation domain, including *TIMP1*, *TAGLN*, and *FLNA* (Fig. 2).

### Transcriptional modules are unique and shared among BPSD domains

**Fig. 3:**
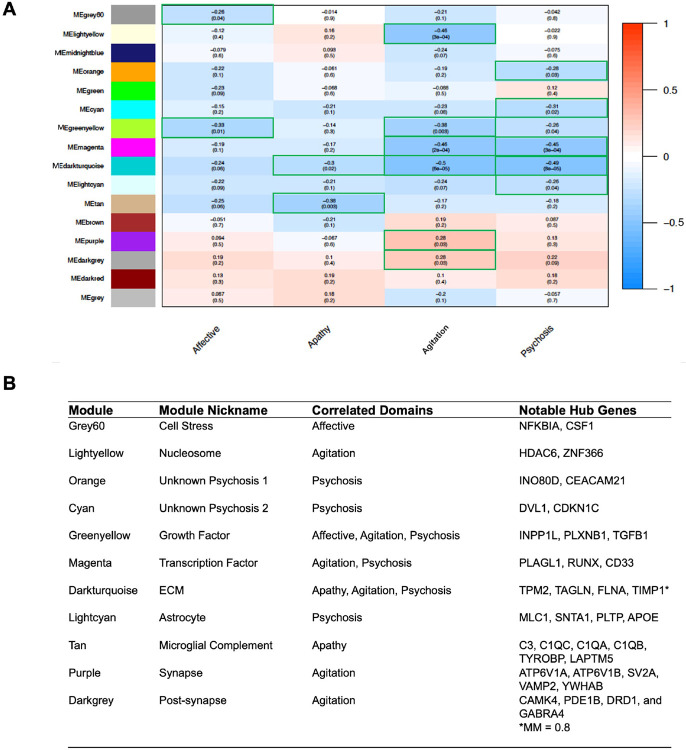
Co-expression modules associated with each BPSD domain. **A)** Heatmap of co-expression modules using WGCNA. Color of each module is arbitrarily chosen, except for the grey module, which represents genes that did not correlate with the expression of other genes. Top number in each square represents the Pearson’s r while the p-value is in the parenthesis underneath. Red color squares have a positive correlation with BPSD status while blue squares have a negative correlation, and the intensity of shading scales with increasing Pearson’s r. **B)** Table of module names, nicknames, correlated domains (p < 0.05), and notable hub genes. All notable hub genes had module membership (MM) > 0.80, except where specified. Gene nicknames were chosen based on the predominant pathways implicated in the functional enrichment analyses.

Genes that are transcribed similarly often regulate similar biological processes, and it can be informative to group genes into co-expression modules to suggest co-regulation. In addition, these co-expression analyses can yield genes that are highly connected to the rest of the network, termed hub genes, that may be central to the transcriptional network and therefore of high interest mechanistically. We utilized WGCNA across all transcriptomes and correlated which modules were linked with the case/control condition for each BPSD domain. As expected, each BPSD domain had some modules shared across certain domains, especially for psychosis and agitation, and other modules that were uniquely significant for a single domain (Fig. 3A). In particular, an 88-gene module for growth factor and cell adhesion (greenyellow) was shared across the affective, agitation, and psychosis domains; a 28-gene module for ECM and actin cytoskeleton (darkturquoise) was shared across apathy, agitation, and psychosis; and a 98-gene module for factor activity (magenta) was shared across agitation and psychosis; no module was associated with all four domains, again suggesting the separability of these traits on a transcriptional level. All shared modules suggested a reduction in transcription in cases, consistent with differential expression trends. Focusing on the agitation domain, three modules were uniquely correlated: modules for nucleosome assembly (lightyellow; 35 genes), ATPase and synaptic signaling (purple; 916 genes), and post-synaptic signaling and response to monoamines (darkgrey; 63-gene). The post-synaptic and monoamine module was enriched for serotoninergic signaling, dopaminergic signaling, and GABAergic signaling. Interestingly, the synaptic signaling modules (purple and darkgrey) were amongst the only significantly correlated modules that demonstrated increased transcription for cases.

Hub genes in WGCNA that are connected with a given domain are defined by the high connectivity within their module as well as significant influence on transcription with the BPSD domain (Fig. 3B). The ECM module, associated strongly with agitation and psychosis and more weakly with apathy, yielded 10 potential hub genes, including *TAGLN* (M_FC,agitation_ = 0.362, p = 1.3 x 10^−5^) and FLNA (M_FC, agitation_ = 0.646, p = 5.9 x 10^−6^). *TIMP1* was also in this domain but had module membership value slightly below the cut-off for potential hub genes. The synaptic signaling and monoamine module (darkgrey), a unique module with increased transcription for aggression domain cases, had 39 hubs genes including *CAMK4* (M_FC, agitation_ = 1.29, p = 0.022), *PDE1B* (M_FC, agitation_ = 1.39, p = 0.007), *DRD1* (M_FC, agitation_ = 1.38, p = 0.006), and *GABRA4* (M_FC, agitation_ = 1.26, p = 0.019).

### Potential drivers of transcriptional network information flow with agitation domain

BPSD likely arise from complex changes in multiple biological networks, and the dynamic interactions of genes and proteins within a network may be key to understanding the difference between the BPSD cases and controls in AD. While enrichment analyses suggest important pathways based on overrepresentation of genes compared to chance, they cannot inform how biological information may change in a context-dependent manner – such as a disease vs non-disease state. Therefore, we used NetDecoder to compare the ‘information-flow’ through each BPSD domain’s cases and controls’ networks, which utilizes a process-guided flow algorithm to identify the weights of information flow from source genes, DEGs, to target genes, transcriptional regulators[48]. For context, when this algorithm was applied to an older breast cancer dataset, they were able to identify three later-confirmed, prognostic markers as important drivers of information flow in the cancer network that were not originally implicated in the original study that lacked a context dependent approach. Important genes in NetDecoder are labeled as high impact genes, network routers, or key targets, and by function of the algorithm, all are genes that were not identified as DEGs but likely affect overall information-flow through the biological system via regulation of downstream transcription.

**Fig. 4:**
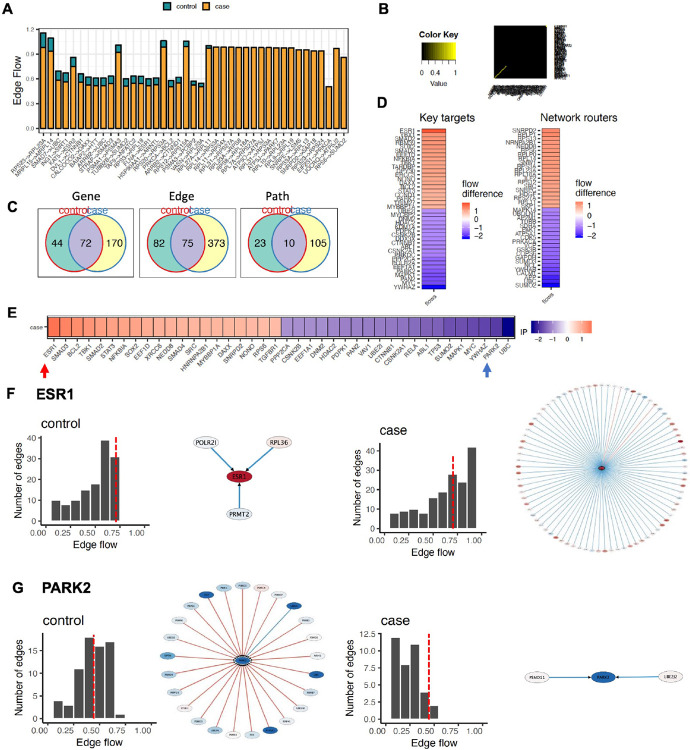
Divergent information flow between transcriptional networks in the agitation domain. **A)** Total edge flow profiles in control versus case subnetworks for the agitation domain. Overall, edges display decreased flow in cases versus controls. **B)** Jaccard index evaluating similarity between case and control subnetworks. **C)** Venn diagrams depicting overlap (blue) in genes, edges, and paths between cases (yellow) and controls (green). **D)** Heatmaps showing top 20 network routers and key targets with the most increased (red) and most decreased (blue) flow difference. Network routers are intermediary genes with the highest difference in flow when comparing the case and control subnetworks. Key targets are sinks, transcriptional regulators in this analysis, that have the highest difference in flow to them when comparing the case and control subnetwork. **E)** Top 20 Impact Genes with the most increased (red) and most decreased (blue) flow differences between case and control subnetwork. Impact scores to determine top impact genes are based on total flow difference at each node between cases and controls, proportion of newly established interaction in the case subnetwork, and number of edges where the expression correlation changes directionality from control to case. Arrows point to notable genes depicted in rest of figure (below) and main text. **F)** Differences in information flow between control and case subnetworks at *ESR1* and **G)** *PARK2*. Node is colored according to the node flow differences across case and control subnetworks. Edge thickness, detailed in histogram on x-axis, represents total amount of edge flow from 0 to 1. The direction of flow is determined by overall structure of information flow from source to sinks. A red edge depicts a positive gene expression correlation between a pair of protein-protein interactions, while blue edges represent a negative correlation. Histogram depicts number of edges binned by total flow. Genes with flow amounts to the right of the dashed, red line are depicted in the corresponding graphic to the right of each histogram.

Focusing on the agitation domain, NetDecoder revealed divergent information flow between cases and controls, and the top 40 network routers, key targets, and high impact genes are shown (Fig. 4 A-E; Supplementary Fig. S3). Though a number of high impact genes are of interest, two are particularly notable (Fig. 4 E, Arrows indicated). The ER-beta, encoded by *ESR1*, was identified as the top key target and high impact gene mediating positive information flow (Fig. 4F). While little remains known about *ESR1* function in agitation/aggression in the frontal cortex, *ESR1*^+^ cells within the hypothalamus and other limbic regions have deep evidence linking them to control of aggressive behaviors[52-57]. The other is Parkin, the ubiquitin ligase encoded by *PARK2* (Fig. 4G) and genetically associated with familial Parkinson’s disease. *PARK2* has significant cross-talk with tau, regulates mitophagy in AD, and interestingly has been linked to impulsive behaviors in Parkinson’s disease[58-60].

## DISCUSSION

The data presented here represents the first exploration of affective, apathy, and agitation symptoms on the transcriptome-wide level and establishes unique patterns of mRNA expression in one of the most consistently implicated brain regions to BPSD, the ACC[49-51]. In addition, we add to the growing knowledge base about transcriptional changes in AD with psychosis[28] (discussed in greater detail along with the affective and apathy domain in supplementary materials). We confirmed that commonly co-occurring BPSD symptoms cluster into domains in our cohort and that these domains are typified by unique transcriptional signatures in the ACC, even when individual samples are used in an overlapping design. Using co-expression analysis, we observed individual BPSD domains being associated with shared and unique transcriptional modules with potential hub genes that may serve as targets for future discovery of discrete molecular mechanisms and novel pharmacology. Finally, we identified key drivers of information flow through biological networks associated with BPSD domains, highlighted by *ESR1* and *PARK2* being potential mediators of the agitation domain in AD.

Though BPSD may be thought of as manifestations of late life primary psychiatric disorders (PPD), there is already some evidence suggesting that BPSD mechanisms diverge molecularly. There have been a handful of genetic studies suggesting overlapping polygenic risk for PPD and neurodegenerative disease[61, 62], and while good epidemiological evidence suggests PPD are risk factors for resultant neurodegenerative dementias[63, 64], the few studies comparing PPD and BPSD on a genetic level have yielded surprising results. For instance, while psychosis – focusing on the ‘positive’ symptoms of hallucinations and delusions – is a prominent symptom in schizophrenia, bipolar disorder, and AD with psychosis, a recent GWAS found negative correlations between schizophrenia and AD with psychosis and bipolar disorder and AD with psychosis, suggesting not just a lack of association between these PPD and BPSD, but a reduced risk of psychosis in AD with increased polygenic risk for schizophrenia or bipolar disorder[65, 66]. This is a stark contrast to the extensive genetic overlap between schizophrenia and bipolar disorder risk[67], suggesting that BPSD may mechanistically distinct from PPD.

Treatment of BPSD has also yielded surprising differences from PPD. For instance, the HTA-SADD trial found no benefit of two common serotonergic antidepressants for depression in AD[68], and a Cochrane Database meta-analysis support this lack of efficacy[69]. Similarly, while selective serotonin reuptake inhibitors (SSRIs) are not used to treat hallucinations and delusions in PPD for psychotic disorders, and may even induce psychosis in bipolar disorder through increasing the risk of mania[70], a common SSRI citalopram seems to have some benefit for reducing these psychotic symptoms in AD, though this was discovered on secondary analysis and requires follow-up[71]. Similar to genetic differences between BPSD and PPD, it seems likely that pharmaceutical approaches need to differ substantially in treating the two disorders, which necessitates the further investigation of BPSD as its own entity distinct from PPD.

Rigorous characterizations of BPSD antemortem with complementary post-mortem tissues for molecular analyses are remarkably scarce, so we sought to optimize our analytical power for the samples we were able to obtain. In addition, given the high prevalence of BPSD in AD ( > 95%), finding enough controls without any BPSD would be very challenging. This required us to adopt an experimental design where each individual’s sample could be considered to be a case (25% highest score of the domain) or control (25% lowest score of the domain) for a specific BPSD domain, meaning that some transcriptome could be potentially analyzed multiple times depending on the comparison. Therefore, it was encouraging to see such a large number of DEGs that are unique to each domain despite the overlapping design, which may suggest that each BPSD domain has distinct biological etiologies despite the common neuropathological drivers – in this case, AD. These findings may be comparable to other investigations showing distinct pathways associated with cognitive decline in AD at the transcriptomic, proteomic, and methylation levels in brain tissue without overlapping dependence on being correlated with AD pathology [72-79], highlighting the heterogeneity of downstream molecular processes from what is putatively considered the upstream etiological agents, namely Aβ and tau. Though this design has the advantage of increasing statistical power in exploring a wide array of symptoms, it presumes separability that precludes identification of individuals with overlapping psychiatric domains that may have unique molecular mechanisms distinct from if these symptoms presented separately. Future investigations can help clarify this.

Given this overlapping design, it was surprising that so few genes were shared amongst all the BPSD domains. It is notable that response to cytokines was implicated as a functionally enriched pathway, as neuroinflammation is often considered an integral driver of neurodegeneration and subsequent synaptic dysfunction[80]. *TIMP1* was among the 22 shared DEGs, all of which were downregulated. A major inhibitor to a number of metalloproteases such as MMP3 and MMP9, *TIMP1* has been implicated in multiple forms of neurodegeneration and neuroinflammation[81, 82], with the hypothesis that early upregulation of *TIMP1* maintains balance in neurodegenerative states while late downregulation may suggest inability to achieve homeostasis[83]. It is therefore speculative but possible that reduced expression of *TIMP1* leads to loss of homeostasis, which could lead to heightened stochasticity in downstream processes and divergent BPSD. While it is also interesting that *TIMP1* has been suggested as a biomarker in biofluids in both Parkinson’s disease and AD[84-86], further exploration of *TIMP1* could be especially fruitful in understanding the early drivers of BPSD.

Though our analyses discovered multiple interesting targets that were unique to each BPSD domain (see supplementary materials for in depth results and discussion), we focused on the results of the agitation domain. The enrichment analysis of the DEGs for this domain were highly suggestive of changes in the ECM or matrisome, and a shared transcriptional module associated with agitation, apathy, and psychosis similarly was enriched for the ECM. A recent and extensive proteome-wide study in AD found that a module of co-expressed proteins enriched for the matrisome was highly correlated with global AD pathology and ApoE status but shockingly independent of cognition[87]. The possibility exists that changes in the ECM are less correlated with cognition but are better tied to BPSD, especially agitation. Interestingly, a common functional SNP in MMP9, which is inhibited by *TIMP1*, was associated with inhibition of aggression and irritability in one study[88]. Currently, much more evidence would be needed to link changes in the ECM with agitation in AD, but this finding further highlights the necessity of considering BPSD in studies of neurodegeneration in addition to cognition, as there may be diverging molecular mechanism related to both sets of symptoms.

The a module for post-synaptic signaling and monoamines was upregulated in agitation cases versus controls and was enriched genes that are common treatment targets for agitation, such as those related to dopamine (antipsychotics), serotonin (antipsychotics and antidepressants), and GABA-A receptors (benzodiazepines)[22]. As monoaminergic treatments are so important in PPD but have limited to no success for the affective domains[68, 69], it was interesting to note that none of the serotonergic, dopaminergic, adrenergic, or muscarinic receptors were DEGs for the affective domain. In contrast, *DRD1* and *5HTR2C* were DEGs for the psychosis and agitation domains while the psychosis domain also demonstrated *DRD2* and *DRD4* as DEGs. While far from conclusive, this again dovetails with the treatment failures for SSRIs for affective behavior in BPSD and further supports the hypothesis that PPD have fewer mechanistic similarities to BPSD.

Additionally, the finding that DRD1, a GPCR coupled to Gs/α, and downstream effectors PDE1B and CAMK4 are associated with this module and the agitation domain is in line with some genetic reports that *DRD1* SNPs are associated with greater impulsivity and aggression[89, 90], including in AD[91, 92] and Parkinson’s disease[93]. The role of striatal role of DRD1 in aggression has been demonstrated before[94], but how DRD1 and some of its downstream signaling molecules affect agitation/aggression in the ACC is less clear. Similar to DRD1, increased GABA-A signaling in the prefrontal cortex, including the ACC, has been linked with increased aggressive and impulsive behaviors[95, 96] and may interact with CAMK4 expression in certain situations[97]. It was notable that those with significant agitation domain behaviors trended towards having more neurons in the ACC than controls, though this analysis precluded investigation of the neuronal subtypes. It is possible that relative increases in GABAergic neurons in cases or decreases in glutamatergic neurons in controls could lead to these differences in agitation domain behavior.

Transcriptome datasets are inherently noisy, and predicting how biological networks regulating transcription, translation, and protein-protein interactions might change based on differential gene expression can uncover hidden drivers of disease. Using an analytical framework to analyze transcription in this context-dependent way, we uncovered two high interest gene targets related to the agitation domain, *ESR1* and *PARK2*. For neurons in the ventromedial hypothalamus[52], posterior and medial amygdala[56], and bed nucleus of the stria terminalis[57], *ESR1*^+^ expression differentiates these cells as ones that regulate aggression from the behavioral function of *ESR1*^−^ cells. Despite the importance of *ESR1* as a marker of aggression-regulating neurons, the actual function of *ESR1* in the cell and resultant aggressive behavior is less clear, though knockout of *ESR1* in mice leads to reduced aggression[98] and *ESR1* polymorphisms have been linked with aggression in humans[99] and songbirds[100]. Understanding *ESR1*’s role in the ACC in terms of agitation, aggression, and impulsivity may be a particularly fruitful avenue for mechanistic understanding and future drug development.

The main novelty of our study is that it is the first, to our knowledge, to study the affective, apathy, and agitation domains on the transcriptome-wide level, which is comparable to the many studies of cognition in AD[73, 75, 77], dating back at least as early as 2008[72], and the recent study of AD with psychosis[28]. Despite this, there are important limitations worth noting. First, our bulk tissue approach precludes deeper exploration of the role of different cell types in BPSD. Similarly, we present only one brain region’s worth of data, and it is likely that interactions with other regions are necessary to fully understand each BPSD domain’s pathogenesis. We were also unable to correlate our findings with medication data before the patient’s death, which will be an important covariate to include in future studies. Additionally, transcriptomic differences do not always translate into protein differences[87], and so future proteomic studies would help solidify the significance of our results. Even with these limitations in mind, we hope our work will lead to future molecular investigations into BPSD so that advanced therapeutics can be designed and translated to the clinic for many of our society’s most vulnerable and affected patients.

## Supplementary Material

Supplementary Table S1 Supplementary Table S1 Questions and evaluations for behavior and psychological symptoms in AD patients with dementia

Supplementary Table S2 **Supplementary Table S2 Notable Affective DEG Enrichments.** Using gProfiler2, functional enrichment analysis was performed, and the most notable pathways are listed in this table. Source indicates the databases queried and include Gene ontology: Molecular function (GO:MF), GO: Biological process (GO:BP), GO: Cellular compartment (GO:CC), Kyoto encyclopedia of gene and genomes (KEGG), and Reactome (REAC). Adjusted p-values (P Adj) > 0.05 were considered significant.

Supplementary Table S3 **Supplementary Table S3 Notable Apathy DEG Enrichments.** Using gProfiler2, functional enrichment analysis was performed, and the most notable pathways are listed in this table. Source indicates the databases queried and include Gene ontology: Molecular function (GO:MF), GO: Biological process (GO:BP), GO: Cellular compartment (GO:CC), Kyoto encyclopedia of gene and genomes (KEGG), Reactome (REAC), WikiPathways (WP), CORUM, and Human phenotype ontology (HP). Adjusted p-values (P Adj) > 0.05 were considered significant.

Supplementary Table S4 **Supplementary Table S4 Notable Psychosis DEG Enrichments.** Using gProfiler2, functional enrichment analysis was performed, and the most notable pathways are listed in this table. Source indicates the databases queried and include Gene ontology: Molecular function (GO:MF), GO: Biological process (GO:BP), GO: Cellular compartment (GO:CC), Kyoto encyclopedia of gene and genomes (KEGG), and Reactome (REAC). Adjusted p-values (P Adj) > 0.05 were considered significant.

supplementary materials

6 **Supplementary Fig.S1 BPSD can be split into four domains based on clustering of symptoms. A)** Principal component analysis was performed on the symptom questions from the structured interview for each participant, and a Scree plot was constructed to evaluate how many factors optimally grouped BPSD. The curve began to asymptote between 3-5 factors. **B**) Horn’s parallel analysis suggested a 4-factor solution, where eigenvalues > 1. **C)** Each BPSD question was loaded onto a predominant factor, and these loadings were used to sort each question into BPSD domains. **D,E)** Variance explained by each factor and correlations between factors.
**Supplementary Fig. S2 Estimated cell abundance for affective, apathy, and psychosis domains. A)** Relative cell abundance estimated with BRETIGIA for affective, **B)** apathy, **C)** and psychosis domains. After correcting for multiple comparisons, there was a significant decrease in microglia detected in Apathy domain cases (p < 0.05) but not for any other comparison.
**Supplementary Fig. S3 Agitation domain case subnetwork.** Main differences in case subnetwork compared to control subnetwork. Source genes are indicated as diamonds, intermediate genes as circles, and target genes as squares. The color of the node depicts flow difference, with red being high flow in case but low in control and blue being high flow in control but low in case. Edge colors correspond to directionality of correlation, with red being positive and blue being negative. To aid visualization, nodes and edges with highest differences in information flow are shown.
**Supplementary Fig. S4 Divergent information flow between transcriptional networks in the affective domain. A)** Total edge flow profiles in control versus case subnetworks for the affective domain. Overall, edges display decreased flow in cases versus controls. **B)** Jaccard index evaluating similarity between case and control subnetworks. **C)** Venn diagrams depicting overlap (blue) in genes, edges, and paths between cases (yellow) and controls (green). **D)** Heatmaps showing top 20 network routers and key targets with the most increased (red) and most decreased (blue) flow difference. Network routers are intermediary genes with the highest difference in flow when comparing the case and control subnetworks. Key targets are sinks, transcriptional regulators in this analysis, that have the highest difference in flow to them when comparing the case and control subnetwork. **E)** Top 20 impact genes with the most increased (red) and most decreased (blue) flow differences between case and control subnetwork. Impact scores to determine top impact genes are based on total flow difference at each node between cases and controls, proportion of newly established interaction in the case subnetwork, and number of edges where the expression correlation changes directionality from control to case. Arrows point to notable genes depicted in rest of figure (below) and main text. **F)** Differences in information flow between control and case subnetworks at *TGFBR1*, *G) TGFBRAP1*, and **H)** *GSK3B*. Node is colored according to the node flow differences across case and control subnetworks. Edge thickness, detailed in histogram on x-axis, represents total amount of edge flow from 0 to 1. The direction of flow is determined by overall structure of information flow from source to sinks. A red edge depicts a positive gene expression correlation between a pair of protein-protein interactions, while blue edges represent a negative correlation. Histogram depicts number of edges binned by total flow. Genes with flow amounts to the right of the dashed, red line are depicted in the corresponding graphic to the right of each histogram.
**Supplementary Fig. S5 Affective domain case subnetwork.** Main differences in case subnetwork compared to control subnetwork. Source genes are indicated as diamonds, intermediate genes as circles, and target genes as squares. The color of the node depicts flow difference, with red being high flow in case but low in control and blue being high flow in control but low in case. Edge colors correspond to directionality of correlation, with red being positive and blue being negative. To aid visualization, nodes and edges with highest differences in information flow are shown.
**Supplementary Fig. S6 Divergent information flow between transcriptional networks in the apathy domain. A)** Total edge flow profiles in control versus case subnetworks for the apathy domain. Overall, edges display decreased flow in cases versus controls. **B)** Jaccard index evaluating similarity between case and control subnetworks. **C)** Venn diagrams depicting overlap (blue) in genes, edges, and paths between cases (yellow) and controls (green). **D)** Heatmaps showing top 20 network routers and key targets with the most increased (red) and most decreased (blue) flow difference. Network routers are intermediary genes with the highest difference in flow when comparing the case and control subnetworks. Key targets are sinks, transcriptional regulators in this analysis, that have the highest difference in flow to them when comparing the case and control subnetwork. **E)** Top 20 Impact Genes with the most increased (red) and most decreased (blue) flow differences between case and control subnetwork. Impact scores to determine top impact genes are based on total flow difference at each node between cases and controls, proportion of newly established interaction in the case subnetwork, and number of edges where the expression correlation changes directionality from control to case. Arrows point to notable genes depicted in rest of figure (below) and main text. **F)** Differences in information flow between control and case subnetworks at *TRAF6*, **G)** *CUL1*, and **H)** *CREBBP*. Node is colored according to the node flow differences across case and control subnetworks. Edge thickness, detailed in histogram on x-axis, represents total amount of edge flow from 0 to 1. The direction of flow is determined by overall structure of information flow from source to sinks. A red edge depicts a positive gene expression correlation between a pair of protein-protein interactions, while blue edges represent a negative correlation. Histogram depicts number of edges binned by total flow. Genes with flow amounts to the right of the dashed, red line are depicted in the corresponding graphic to the right of each histogram.
**Supplementary Fig. S7 Apathy domain case subnetwork.** Main differences in case subnetwork compared to control subnetwork. Source genes are indicated as diamonds, intermediate genes as circles, and target genes as squares. The color of the node depicts flow difference, with red being high flow in case but low in control and blue being high flow in control but low in case. Edge colors correspond to directionality of correlation, with red being positive and blue being negative. To aid visualization, nodes and edges with highest differences in information flow are shown.
**Supplementary Fig. S8 Divergent information flow between transcriptional networks in the psychosis domain. A)** Total edge flow profiles in control versus case subnetworks for the Psychosis Domain. Overall, edges display decreased flow in cases versus controls. **B)** Jaccard index evaluating similarity between case and control subnetworks. **C)** Venn diagrams depicting overlap (blue) in genes, edges, and paths between cases (yellow) and controls (green). **D)** Heatmaps showing top 20 network routers and key targets with the most increased (red) and most decreased (blue) flow difference. Network routers are intermediary genes with the highest difference in flow when comparing the case and control subnetworks. Key targets are sinks, transcriptional regulators in this analysis, that have the highest difference in flow to them when comparing the case and control subnetwork. **E)** Top 20 Impact Genes with the most increased (red) and most decreased (blue) flow differences between case and control subnetwork. Impact scores to determine top impact genes are based on total flow difference at each node between cases and controls, proportion of newly established interaction in the case subnetwork, and number of edges where the expression correlation changes directionality from control to case. Arrows point to notable genes depicted in rest of figure (below) and main text. **F)** Differences in information flow between control and case subnetworks at *HSP90AA1*, **G)** *ARRB1*, and **H)** *YWHAZ*. Node is colored according to the node flow differences across case and control subnetworks. Edge thickness, detailed in histogram on x-axis, represents total amount of edge flow from 0 to 1. The direction of flow is determined by overall structure of information flow from source to sinks. A red edge depicts a positive gene expression correlation between a pair of protein-protein interactions, while blue edges represent a negative correlation. Histogram depicts number of edges binned by total flow. Genes with flow amounts to the right of the dashed, red line are depicted in the corresponding graphic to the right of each histogram.
**Supplementary Fig. S9 Notable key target and network router for psychosis domain.** Despite not affecting enough information flow to be considered high impact genes, a notable key target in *HTT* (**A)** and a notable network router in SCNA (**B**). *HTT* encodes huntingtin, which is the protein implicated in the neurodegenerative condition Huntington’s Disease. There is a notable shift of information flow away from the post-synaptic protein *DLG4* (PSD95) in the control condition towards proteins involved in the unfolded protein response. *SCNA* encodes α-synuclein, which is the protein implicated in the neurodegenerative disease Parkinson’s disease (**B, C**).
**Supplementary Fig. S10 Psychosis domain case subnetwork.** Main differences in case subnetwork compared to control subnetwork. Source genes are indicated as diamonds, intermediate genes as circles, and target genes as squares. The color of the node depicts flow difference, with red being high flow in case but low in control and blue being high flow in control but low in case. Edge colors correspond to directionality of correlation, with red being positive and blue being negative. To aid visualization, nodes and edges with highest differences in information flow are shown.
**Supplementary Fig. S11 WGCNA optimization and module dendrogram. A)** Using the Abbassi-Daloii WGCNA optimization method, 420 Enrichment factors (EF) were computed based on corresponding differences in WGCNA parameters. These EFs were plotted against the number of modules with a weak linear correlation. The module circled in red depicts the EF of the parameters ultimately chosen for WGCNA, as this was both the highest EF and resulted in a moderate number of modules. **B)** Network properties for different soft thresholds visualized by graphing power by scale free topology fit. Notably, network is signed. Mean connectivity at different powers is also shown. **C)** Module dendrogram depicting clustering of genes into modules based on high co-expression.

## Figures and Tables

**Table 1.** RNA-seq sampling information of cases and controls for different BPSD domains in AD subjects

Affective Domain				
	Control (n=22)	Case (n=21)	Statistical Test	P-value (α=0.05)
**Female Gender** n, (%)				
Female	12 (54.55%)	9 (42.86%)	Fisher's exact test	NS
**Age at Death** (years)				
M (SD)	81.24 (7.58)	81.07 (8.31)	Welch's t=0.07 (df=40.22)	NS
**Braak Stage** n, (%)				
IV	2 (9.09%)	3 (14.29%)	χ^2^(2)=1.97	NS
V	8 (36.36%)	11 (52.38%)		
VI	12 (54.55%)	7 (33.33%)		
**CERAD Score** n, (%)				
2	3 (13.64%)	1 (4.76%)	Fisher's exact test	NS
1	19 (86.36%)	20 (95.24%)		
**NIA Raegan Score** n, (%)				
2	5 (22.73%)	3 (14.29%)	Fisher's exact test	NS
1	17 (77.27%)	18 (85.71%)		
**PMI (Hours)**				
M (SD)	5.01 (1.67)	6.51 (5.36)	Welch's t=1.23 (df=23.71)	NS
Apathy Domain				
	Control (n=23)	Case (n=24)	Statistical Test	P-value (α=0.05)
**Female Gender** n, (%)				
Female	12 (52.17%)	13 (54.17%%)	Fisher's exact test	NS
**Age at Death** (years)				
M (SD)	81.90 (8.83)	79.51 (8.13)	Welch's t=0.96 (df=44.31)	NS
**Braak Stage** n, (%)				
IV	4 (17.39%)	1 (4.17%)	χ^2^(2)=6.62	p<0.05
V	7 (30.43%)	16 (66.67%)		
VI	12 (52.17%)	7 (29.17%)		
**CERAD Score** n, (%)				
2	4 (17.39%)	2 (8.33%)	Fisher's exact test	NS
1	19 (82.61%)	22 (91.67%)		
**NIA Raegan Score** n, (%)				
2	6 (26.09%)	3 (12.50%)	Fisher's exact test	NS
1	17 (73.91%)	21 (87.50%)		
**PMI (Hours)**				
M (SD)	7.10 (5.59)	5.94 (3.18)	Welch's t=0.87 (df=34.57)	NS
Agitation Domain				
	Control (n=27)	Case (n=22)	Statistical Test	P-value (α=0.05)
**Female Gender** n, (%)				
Female	14 (51.85%)	10 (45.45%)	Fisher's exact test	NS
**Age at Death** (years)				
M (SD)	81.00 (7.56)	78.71 (7.76)	Welch's t=1.04 (df=44.59)	NS
**Braak Stage** n, (%)				
IV	4 (14.81%)	2 (9.09%)	χ^2^(2)=0.39	NS
V	12 (44.44%)	10 (45.45%)		
VI	11 (40.74%)	10 (45.45%)		
**CERAD Score** n, (%)				
2	4 (14.81%)	1 (4.54%)	Fisher's exact test	NS
1	23 (85.19%)	21 (95.45%)		
**NIA Raegan Score** n, (%)				
2	7 (25.93%)	2 (9.09%)	Fisher's exact test	NS
1	20 (74.07%)	20 (90.91%)		
**PMI (Hours)**				
M (SD)	6.96 (5.01)	5.33 (2.10)	Welch's t=1.53 (df=36.30)	NS
Psychosis Domain				
	Control (n=25)	Case (n=20)	Statistical Test	P-value (α=0.05)
**Female Gender** n, (%)				
Female	13 (52%)	10 (50%)	Fisher's exact test	NS
**Age at Death** (years)				
M (SD)	82.18 (7.62)	78.91 (7.09)	Welch's t=1.49 (df=41.98)	NS
**Braak Stage** n, (%)				
IV	3 (12%)	1 (5%)	χ^2^(2)=0.72	NS
V	12 (48%)	11 (55%)		
VI	10 (40%)	8 (40%)		
**CERAD Score** n, (%)				
2	4 (16%)	0 (0%)	Fisher's exact test	NS
1	21 (84%)	20 (100%)		
**NIA Raegan Score** n, (%)				
2	7 (28%)	1 (5%)	Fisher's exact test	NS (p=0.06)
1	18 (72%)	19 (95%)		
**PMI (Hours)**				
M (SD)	6.10 (2.67)	6.12 (5.54)	Welch's t=0.01 (df=26.01)	NS

## References

[1] Rog LA, Park LQ, Harvey DJ, Huang C-J, Mackin S, Farias ST. The Independent Contributions of Cognitive Impairment and Neuropsychiatric Symptoms to Everyday Function in Older Adults. Clin Neuropsychol. 2014;28:215–236. 24502686 10.1080/13854046.2013.876101 PMC4021718

[2] Vik-Mo AO, Giil LM, Ballard C, Aarsland D. Course of neuropsychiatric symptoms in dementia: 5-year longitudinal study. Int J Geriatr Psychiatry. 2018;33:1361–1369. 29979473 10.1002/gps.4933

[3] Schwertner E, Pereira JB, Xu H, Secnik J, Winblad B, Eriksdotter M, Behavioral and Psychological Symptoms of Dementia in Different Dementia Disorders: A Large-Scale Study of 10,000 Individuals. J Alzheimers Dis JAD. 2022;87:1307–1318. 35491774 10.3233/JAD-215198 PMC9198804

[4] Taragano F, Allegri R, Krupitzki H, Sarasola D, Serrano C, Loñ L, Mild behavioral impairment and risk of dementia. J Clin Psychiatry. 2009;70:584–592. 19323967 10.4088/jcp.08m04181 PMC2711522

[5] Ismail Z, Smith EE, Geda Y, Sultzer D, Brodaty H, Smith G, Neuropsychiatric symptoms as early manifestations of emergent dementia: Provisional diagnostic criteria for mild behavioral impairment. Alzheimers Dement J Alzheimers Assoc. 2016;12:195–202. 10.1016/j.jalz.2015.05.017 PMC4684483 26096665

[6] Geda YE, Roberts RO, Mielke MM, Knopman DS, Christianson TJH, Pankratz VS, Baseline Neuropsychiatric Symptoms and the Risk of Incident Mild Cognitive Impairment: A Population-Based Study. Am J Psychiatry. 2014;171:572–581. 24700290 10.1176/appi.ajp.2014.13060821 PMC4057095

[7] Wise EA, Rosenberg PB, Lyketsos CG, Leoutsakos J-M. Time course of neuropsychiatric symptoms and cognitive diagnosis in National Alzheimer’s Coordinating Centers volunteers. Alzheimers Dement Diagn Assess Dis Monit. 2019;11:333–339. 10.1016/j.dadm.2019.02.006 PMC6476801 31024987

[8] Burhanullah MH, Tschanz JT, Peters ME, Leoutsakos J-M, Matyi J, Lyketsos CG, Neuropsychiatric Symptoms as Risk Factors for Cognitive Decline in Clinically Normal Older Adults: The Cache County Study. Am J Geriatr Psychiatry. 2020;28:64–71. 31186157 10.1016/j.jagp.2019.03.023 PMC6874723

[9] Pietrzak RH, Lim YY, Neumeister A, Ames D, Ellis KA, Harrington K, Amyloid-β, anxiety, and cognitive decline in preclinical Alzheimer disease: a multicenter, prospective cohort study. JAMA Psychiatry. 2015;72:284–291. 25629787 10.1001/jamapsychiatry.2014.2476

[10] Leoutsakos J-MS, Forrester SN, Lyketsos CG, Smith GS. Latent classes of neuropsychiatric symptoms in NACC controls and conversion to MCI or dementia. J Alzheimers Dis JAD. 2015;48:483–493. 26402012 10.3233/JAD-150421 PMC4635658

[11] Roberto N, Portella MJ, Marquié M, Alegret M, Hernández I, Mauleón A, Neuropsychiatric Profile as a Predictor of Cognitive Decline in Mild Cognitive Impairment. Front Aging Neurosci. 2021;13:718949. 34955804 10.3389/fnagi.2021.718949 PMC8693625

[12] David ND, Lin F, Porsteinsson AP. Trajectories of neuropsychiatric symptoms and cognitive decline in mild cognitive impairment. Am J Geriatr Psychiatry Off J Am Assoc Geriatr Psychiatry. 2016;24:70–80. 10.1016/j.jagp.2015.06.001 PMC4691566 26525995

[13] Jang JY, Ho JK, Blanken AE, Dutt S, Nation DA. Affective neuropsychiatric symptoms as early signs of dementia risk in older adults. J Alzheimers Dis JAD. 2020;77:1195–1207. 32925031 10.3233/JAD-200190 PMC8034499

[14] Teng E, Lu PH, Cummings JL. Neuropsychiatric Symptoms Are Associated with Progression from Mild Cognitive Impairment to Alzheimer’s Disease. Dement Geriatr Cogn Disord. 2007;24:253–259. 17700021 10.1159/000107100

[15] Peters ME, Schwartz S, Han D, Rabins PV, Steinberg M, Tschanz JT, Neuropsychiatric Symptoms as Predictors of Progression to Severe Alzheimer’s Dementia and Death: The Cache County Dementia Progression Study. Am J Psychiatry. 2015;172:460–465. 25585033 10.1176/appi.ajp.2014.14040480 PMC4416978

[16] Buckley T, Fauth EB, Morrison A, Tschanz J, Rabins PV, Piercy KW, Predictors of Quality of Life Ratings for Persons with Dementia Simultaneously Reported by Patients and their Caregivers: The Cache County (Utah) Study. Int Psychogeriatr IPA. 2012;24:1094–1102. 10.1017/S1041610212000063 PMC3523699 22414494

[17] Burks HB, Bordes JKA des, Chadha R, Holmes HM, Rianon NJ. Quality of Life Assessment in Older Adults with Dementia: A Systematic Review. Dement Geriatr Cogn Disord. 2021;50:103–110. 34167127 10.1159/000515317

[18] Kaufer DI, Cummings JL, Christine D, Bray T, Castellon S, Masterman D, Assessing the impact of neuropsychiatric symptoms in Alzheimer’s disease: the Neuropsychiatric Inventory Caregiver Distress Scale. J Am Geriatr Soc. 1998;46:210–215. 9475452 10.1111/j.1532-5415.1998.tb02542.x

[19] Cheng S-T. Dementia Caregiver Burden: a Research Update and Critical Analysis. Curr Psychiatry Rep. 2017;19:64. 28795386 10.1007/s11920-017-0818-2 PMC5550537

[20] Backhouse T, Camino J, Mioshi E. What Do We Know About Behavioral Crises in Dementia? A Systematic Review. J Alzheimers Dis. 2018;62:99–113. 29439334 10.3233/JAD-170679

[21] Verbeek H, Meyer G, Challis D, Zabalegui A, Soto ME, Saks K, Inter-country exploration of factors associated with admission to long-term institutional dementia care: evidence from the RightTimePlaceCare study. J Adv Nurs. 2015;71:1338–1350. 25869186 10.1111/jan.12663

[22] Keszycki RM, Fisher DW, Dong H. The Hyperactivity-Impulsivity-Irritiability-Disinhibition-Aggression-Agitation Domain in Alzheimer’s Disease: Current Management and Future Directions. Front Pharmacol. 2019;10:1109. 31611794 10.3389/fphar.2019.01109 PMC6777414

[23] Robert PH, Verhey FRJ, Byme EJ, Hurt C, Deyn PPD, Nobili F, Grouping for behavioral and psychological symptoms in dementia: clinical and biological aspects. Consensus paper of the European Alzheimer disease consortium. Eur Psychiatry. 2005;20:490–496. 16310680 10.1016/j.eurpsy.2004.09.031

[24] van der Linde RM, Dening T, Matthews FE, Brayne C. Grouping of behavioural and psychological symptoms of dementia. Int J Geriatr Psychiatry. 2014;29:562–568. 24677112 10.1002/gps.4037 PMC4255309

[25] Hirao K, Pontone GM, Smith GS. Molecular Imaging of Neuropsychiatric Symptoms in Alzheimer’s and Parkinson’s disease. Neurosci BiobehavRev. 2015;49:157–170. 10.1016/j.neubiorev.2014.11.010 PMC4806385 25446948

[26] Gotovac K, Nikolac Perković M, Pivac N, Borovečki F. Biomarkers of aggression in dementia. Prog Neuropsychopharmacol Biol Psychiatry. 2016;69:125–130. 26952705 10.1016/j.pnpbp.2016.03.002

[27] Lukiw WJ, Rogaev EL Genetics of Aggression in Alzheimer’s Disease (AD). Front Aging Neurosci. 2017;9:87. 28443016 10.3389/fnagi.2017.00087 PMC5385328

[28] DeChellis-Marks MR, Wei Y, Ding Y, Wolfe CM, Krivinko JM, MacDonald ML, Psychosis in Alzheimer’s Disease Is Associated With Increased Excitatory Neuron Vulnerability and Post-transcriptional Mechanisms Altering Synaptic Protein Levels. Front Neurol. 2022;13:778419. 35309563 10.3389/fneur.2022.778419 PMC8925864

[29] Murray PS, Kumar S, DeMichele-Sweet MAA, Sweet RA. Psychosis in Alzheimer’s Disease. Biol Psychiatry. 2014;75:542–552. 24103379 10.1016/j.biopsych.2013.08.020 PMC4036443

[30] Krivinko JM, Erickson SL, Ding Y, Sun Z, Penzes P, MacDonald ML, Synaptic proteome compensation and resilience to psychosis in Alzheimer Disease. Am J Psychiatry. 2018;175:999–1009. 30021459 10.1176/appi.ajp.2018.17080858 PMC6167138

[31] Gilley DW, Wilson RS, Beckett LA, Evans DA. Psychotic Symptoms and Physically Aggressive Behavior in Alzheimer’s Disease. J Am Geriatr Soc. 1997;45:1074–1079. 9288014 10.1111/j.1532-5415.1997.tb05969.x

[32] Gilley DW, Whalen ME, Wilson RS, Bennett DA. Hallucinations and associated factors in Alzheimer’s disease. J Neuropsychiatry Clin Neurosci. 1991;3:371–376. 1821255 10.1176/jnp.3.4.371

[33] Gilley DW, Wilson RS, Bienias JL, Bennett DA, Evans DA. Predictors of depressive symptoms in persons with Alzheimer’s disease. J Gerontol B Psychol Sci Soc Sci. 2004;59:P75–83. 15014090 10.1093/geronb/59.2.p75

[34] Wilson R, Gilley D, Bennett D, Beckett L, Evans D. Hallucinations, delusions, and cognitive decline in Alzheimer’s disease. J Neurol Neurosurg Psychiatry. 2000;69:172–177. 10896689 10.1136/jnnp.69.2.172 PMC1737043

[35] Blacker D, Albert MS, Bassett SS, Go RC, Harrell LE, Folstein MF. Reliability and validity of NINCDS-ADRDA criteria for Alzheimer’s disease. The National Institute of Mental Health Genetics Initiative. Arch Neurol. 1994;51:1198–1204. 7986174 10.1001/archneur.1994.00540240042014

[36] Consensus recommendations for the postmortem diagnosis of Alzheimer’s disease. The National Institute on Aging, and Reagan Institute Working Group on Diagnostic Criteria for the Neuropathological Assessment of Alzheimer’s Disease. Neurobiol Aging. 1997; 18: S1–2. 9330978

[37] Bennett DA, Schneider JA, Arvanitakis Z, Kelly JF, Aggarwal NT, Shah RC, Neuropathology of older persons without cognitive impairment from two community-based studies. Neurology. 2006;66:1837–1844. 16801647 10.1212/01.wnl.0000219668.47116.e6

[38] Bray NL, Pimentel H, Melsted P, Pachter L. Near-optimal probabilistic RNA-seq quantification. Nat Biotechnol. 2016;34:525–527. 27043002 10.1038/nbt.3519

[39] Li J, Bushel P, Chu T-M, Wolfinger R. Batch Effects and Noise in Microarray Experiments: Sources and Solutions. Chapter 12: Principal Variance Components Analysis: Estimating Batch Effects in Microarray Gene Expression Data. Wiley.

[40] Chen LS, Storey JD. Eigen-R2 for dissecting variation in high-dimensional studies. Bioinforma Oxf Engl. 2008;24:2260–2262. 10.1093/bioinformatics/btn411 PMC4038975 18718946

[41] Love MI, Huber W, Anders S. Moderated estimation of fold change and dispersion for RNA-seq data with DESeq2. Genome Biol. 2014;15:550. 25516281 10.1186/s13059-014-0550-8 PMC4302049

[42] Raudvere U, Kolberg L, Kuzmin I, Arak T, Adler P, Peterson H, g:Profiler: a web server for functional enrichment analysis and conversions of gene lists (2019 update). Nucleic Acids Res. 2019;47:W191–W198. 31066453 10.1093/nar/gkz369 PMC6602461

[43] Ritchie ME, Phipson B, Wu D, Hu Y, Law CW, Shi W, limma powers differential expression analyses for RNA-sequencing and microarray studies. Nucleic Acids Res. 2015;43:e47. 25605792 10.1093/nar/gkv007 PMC4402510

[44] McKenzie AT, Wang M, Hauberg ME, Fullard JF, Kozlenkov A, Keenan A, Brain Cell Type Specific Gene Expression and Co-expression Network Architectures. Sci Rep. 2018;8:8868. 29892006 10.1038/s41598-018-27293-5 PMC5995803

[45] Jew B, Alvarez M, Rahmani E, Miao Z, Ko A, Garske KM, Accurate estimation of cell composition in bulk expression through robust integration of single-cell information. Nat Commun. 2020;11:1971. 32332754 10.1038/s41467-020-15816-6 PMC7181686

[46] Zhang B, Horvath S. A general framework for weighted gene co-expression network analysis. Stat Appl Genet Mol Biol. 2005;4:Article17. 16646834 10.2202/1544-6115.1128

[47] Langfelder P, Horvath S. WGCNA: an R package for weighted correlation network analysis. BMC Bioinformatics. 2008;9:559. 19114008 10.1186/1471-2105-9-559 PMC2631488

[48] da Rocha EL, Ung CY, McGehee CD, Correia C, Li H. NetDecoder: a network biology platform that decodes context-specific biological networks and gene activities. Nucleic Acids Res. 2016;44:e100. 26975659 10.1093/nar/gkw166 PMC4889937

[49] Theleritis C, Politis A, Siarkos K, Lyketsos CG. A review of neuroimaging findings of apathy in Alzheimer’s Disease. Int Psychogeriatr IPA. 2014;26:195–207. 10.1017/S1041610213001725 PMC4086515 24135083

[50] Rosenberg PB, Nowrangi MA, Lyketsos CG. Neuropsychiatric symptoms in Alzheimer’s disease: What might be associated brain circuits? Mol Aspects Med. 2015;0:25–37. 10.1016/j.mam.2015.05.005 PMC4600424 26049034

[51] Tekin S, Mega MS, Masterman DM, Chow T, Garakian J, Vinters HV, Orbitofrontal and anterior cingulate cortex neurofibrillary tangle burden is associated with agitation in Alzheimer disease. Ann Neurol. 2001;49:355–361. 11261510

[52] Lee H, Kim D-W, Remedios R, Anthony TE, Chang A, Madisen L, Scalable control of mounting and attack by Esr1+ neurons in the ventromedial hypothalamus. Nature. 2014;509:627–632. 24739975 10.1038/nature13169 PMC4098836

[53] Hashikawa K, Hashikawa Y, Tremblay R, Zhang J, Feng JE, Sabol A, Esr1+ cells in the ventromedial hypothalamus control female aggression. Nat Neurosci. 2017;20:1580–1590. 28920934 10.1038/nn.4644 PMC5953764

[54] Karigo T, Kennedy A, Yang B, Liu M, Tai D, Wahle IA, Distinct hypothalamic control of same-and opposite-sex mounting behaviour in mice. Nature. 2021;589:258–263. 33268894 10.1038/s41586-020-2995-0 PMC7899581

[55] Sano K, Nakata M, Musatov S, Morishita M, Sakamoto T, Tsukahara S, Pubertal activation of estrogen receptor α in the medial amygdala is essential for the full expression of male social behavior in mice. Proc Natl Acad Sci. 2016;113:7632–7637. 27325769 10.1073/pnas.1524907113 PMC4941494

[56] Yamaguchi T, Wei D, Song SC, Lim B, Tritsch NX, Lin D. Posterior amygdala regulates sexual and aggressive behaviors in male mice. Nat Neurosci. 2020;23:1111–1124. 32719562 10.1038/s41593-020-0675-x PMC7483354

[57] Knoedler JR, Inoue S, Bayless DW, Yang T, Tantry A, Davis C, A functional cellular framework for sex and estrous cycle-dependent gene expression and behavior. Cell. 2022;185:654–671.e22. 35065713 10.1016/j.cell.2021.12.031 PMC8956134

[58] Menéndez J, Rodríguez-Navarro JA, Solano RM, Casarejos MJ, Rodal I, Guerrero R, Suppression of Parkin enhances nigrostriatal and motor neuron lesion in mice over-expressing human-mutated tau protein. Hum Mol Genet. 2006;15:2045–2058. 16698879 10.1093/hmg/ddl129

[59] Bhattacharjee S. Impulse control disorders in Parkinson’s disease: Review of pathophysiology, epidemiology, clinical features, management, and future challenges. Neurol India. 2018;66:967. 30038082 10.4103/0028-3886.237019

[60] Goudarzi S, Hosseini A, Abdollahi M, Haghi-Aminjan H. Insights Into Parkin-Mediated Mitophagy in Alzheimer’s Disease: A Systematic Review. Front Aging Neurosci. 2021;13:674071. 34393755 10.3389/fnagi.2021.674071 PMC8358451

[61] Lutz MW, Sprague D, Barrera J, Chiba-Falek O. Shared genetic etiology underlying Alzheimer’s disease and major depressive disorder. Transl Psychiatry. 2020;10:1–14. 32152295 10.1038/s41398-020-0769-y PMC7062839

[62] Wingo TS, Liu Y, Gerasimov ES, Vattathil SM, Wynne ME, Liu J, Shared mechanisms across the major psychiatric and neurodegenerative diseases. Nat Commun. 2022; 13:4314. 35882878 10.1038/s41467-022-31873-5 PMC9325708

[63] Skogen JC, Bergh S, Stewart R, Knudsen AK, Bjerkeset O. Midlife mental distress and risk for dementia up to 27 years later: the Nord-Trøndelag Health Study (HUNT) in linkage with a dementia registry in Norway. BMC Geriatr. 2015; 15:23. 25886723 10.1186/s12877-015-0020-5 PMC4571744

[64] Richmond-Rakerd LS, D’Souza S, Milne B J, Caspi A, Moffitt TE. Longitudinal Associations of Mental Disorders With Dementia: 30-Year Analysis of 1.7 Million New Zealand Citizens. JAMA Psychiatry. 2022. 16 February 2022. 10.1001/jamapsychiatry.2021.4377. PMC8851362 35171209

[65] DeMichele-Sweet MAA, Weamer EA, Klei L, Vrana DT, Hollingshead DJ, Seltman HJ, Genetic Risk for Schizophrenia and Psychosis in Alzheimer Disease. Mol Psychiatry. 2018;23:963–972. 28461698 10.1038/mp.2017.81 PMC5668212

[66] DeMichele-Sweet MAA, Klei L, Creese B, Harwood JC, Weamer EA, McClain L, Genome-wide association identifies the first risk loci for psychosis in Alzheimer disease. Mol Psychiatry. 2021:1–15. 34112972 10.1038/s41380-021-01152-8 PMC8660923

[67] Cross-Disorder Group of the Psychiatric Genomics Consortium. Identification of risk loci with shared effects on five major psychiatric disorders: a genome-wide analysis. Lancet. 2013;381:1371–1379. 23453885 10.1016/S0140-6736(12)62129-1 PMC3714010

[68] Banerjee S, Hellier J, Dewey M, Romeo R, Ballard C, Baldwin R, Sertraline or mirtazapine for depression in dementia (HTA-SADD): a randomised, multicentre, double-blind, placebo-controlled trial. The Lancet. 2011;378:403–411. 10.1016/S0140-6736(11)60830-1 21764118

[69] Dudas R, Malouf R, McCleery J, Dening T. Antidepressants for treating depression in dementia. Cochrane Database Syst Rev. 2018;2018:CD003944. 10.1002/14651858.CD003944.pub2 PMC6513376 30168578

[70] Tondo L, Vázquez G, Baldessarini RJ. Mania associated with antidepressant treatment: comprehensive meta-analytic review. Acta Psychiatr Scand. 2010;121:404–414. 19958306 10.1111/j.1600-0447.2009.01514.x

[71] Leonpacher AK, Peters ME, Drye LT, Makino KM, Newell JA, Devanand D p., Effects of Citalopram on Neuropsychiatric Symptoms in Alzheimer’s Dementia: Evidence From the CitAD Study. Am J Psychiatry. 2016;173:473–480. 27032628 10.1176/appi.ajp.2016.15020248

[72] Wilmot B, McWeeney SK, Nixon RR, Montine TJ, Laut J, Harrington CA, Translational gene mapping of cognitive decline. Neurobiol Aging. 2008;29:524–541. 17174450 10.1016/j.neurobiolaging.2006.11.008 PMC2684335

[73] Wang M, Roussos P, McKenzie A, Zhou X, Kajiwara Y, Brennand KJ, Integrative network analysis of nineteen brain regions identifies molecular signatures and networks underlying selective regional vulnerability to Alzheimer’s disease. Genome Med. 2016;8:104. 27799057 10.1186/s13073-016-0355-3 PMC5088659

[74] Seyfried NT, Dammer EB, Swarup V, Nandakumar D, Duong DM, Yin L, A Multi-Network Approach Identifies Protein-specific Co-expression in Asymptomatic and Symptomatic Alzheimer’s Disease. Cell Syst. 2017;4:60–72.e4. 27989508 10.1016/j.cels.2016.11.006 PMC5269514

[75] Mostafavi S, Gaiteri C, Sullivan SE, White CC, Tasaki S, Xu J, A molecular network of the aging human brain provides insights into the pathology and cognitive decline of Alzheimer’s disease. Nat Neurosci. 2018;21:811–819. 29802388 10.1038/s41593-018-0154-9 PMC6599633

[76] Li P, Marshall L, Oh G, Jakubowski JL, Groot D, He Y, Epigenetic dysregulation of enhancers in neurons is associated with Alzheimer’s disease pathology and cognitive symptoms. Nat Commun. 2019;10:2246. 31113950 10.1038/s41467-019-10101-7 PMC6529540

[77] Mathys H, Davila-Velderrain J, Peng Z, Gao F, Mohammadi S, Young JZ, Single-cell transcriptomic analysis of Alzheimer’s disease. Nature. 2019;570:332–337. 31042697 10.1038/s41586-019-1195-2 PMC6865822

[78] Patel H, Hodges AK, Curtis C, Lee SH, Troakes C, Dobson RJB, Transcriptomic analysis of probable asymptomatic and symptomatic alzheimer brains. Brain Behav Immun. 2019;80:644–656. 31063847 10.1016/j.bbi.2019.05.009

[79] Wang Q, Chen K, Su Y, Reiman EM, Dudley JT, Readhead B. Deep learning-based brain transcriptomic signatures associated with the neuropathological and clinical severity of Alzheimer’s disease. Brain Commun. 2021;4:fcab293. 34993477 10.1093/braincomms/fcab293 PMC8728025

[80] Heneka MT, Carson MJ, Khoury JE, Landreth GE, Brosseron F, Feinstein DL, Neuroinflammation in Alzheimer’s disease. Lancet Neurol. 2015;14:388–405. 25792098 10.1016/S1474-4422(15)70016-5 PMC5909703

[81] Wang X-X, Tan M-S, Yu J-T, Tan L. Matrix Metalloproteinases and Their Multiple Roles in Alzheimer’s Disease. BioMed Res Int. 2014;2014:908636. 25050378 10.1155/2014/908636 PMC4094696

[82] Behl T, Kaur G, Sehgal A, Bhardwaj S, Singh S, Buhas C, Multifaceted Role of Matrix Metalloproteinases in Neurodegenerative Diseases: Pathophysiological and Therapeutic Perspectives. Int J Mol Sci. 2021;22:1413. 33573368 10.3390/ijms22031413 PMC7866808

[83] Saha P, Sarkar S, Paidi RK, Biswas SC. TIMP-1: A key cytokine released from activated astrocytes protects neurons and ameliorates cognitive behaviours in a rodent model of Alzheimer’s disease. Brain Behav Immun. 2020;87:804–819. 32194232 10.1016/j.bbi.2020.03.014

[84] Shi M, Movius J, Dator R, Aro P, Zhao Y, Pan C, Cerebrospinal fluid peptides as potential Parkinson disease biomarkers: a staged pipeline for discovery and validation. Mol Cell Proteomics MCP. 2015;14:544–555. 25556233 10.1074/mcp.M114.040576 PMC4349976

[85] Yao F, Zhang K, Zhang Y, Guo Y, Li A, Xiao S, Identification of Blood Biomarkers for Alzheimer’s Disease Through Computational Prediction and Experimental Validation. Front Neurol. 2018;9:1158. 30671019 10.3389/fneur.2018.01158 PMC6331438

[86] Lorenzl S, Albers DS, LeWitt PA, Chirichigno JW, Hilgenberg SL, Cudkowicz ME, Tissue inhibitors of matrix metalloproteinases are elevated in cerebrospinal fluid of neurodegenerative diseases. J Neurol Sci. 2003;207:71–76. 12614934 10.1016/s0022-510x(02)00398-2

[87] Johnson ECB, Carter EK, Dammer EB, Duong DM, Gerasimov ES, Liu Y, Large-scale deep multi-layer analysis of Alzheimer’s disease brain reveals strong proteomic disease-related changes not observed at the RNA level. Nat Neurosci. 2022;25:213–225. 35115731 10.1038/s41593-021-00999-y PMC8825285

[88] Suchankova P, Pettersson R, Nordenström K, Holm G, Ekman A. Personality traits and the R668Q polymorphism located in the MMP-9 gene. Behav Brain Res. 2012;228:232–235. 22142952 10.1016/j.bbr.2011.11.026

[89] Pardini M, Krueger F, Hodgkinson CA, Raymont V, Strenziok M, Amore M, Aggression, DRD1 polymorphism, and lesion location in penetrating traumatic brain injury. CNS Spectr. 2014;19:382–390. 24618367 10.1017/S1092852914000108 PMC4161664

[90] Park CI, Kim HW, Hwang SS, Kang JI, Kim SJ. Influence of dopamine-related genes on craving, impulsivity, and aggressiveness in Korean males with alcohol use disorder. Eur Arch Psychiatry Clin Neurosci. 2021;271:865–872. 31559529 10.1007/s00406-019-01072-3

[91] Sweet RA, Nimgaonkar VL, Kamboh MI, Lopez OL, Zhang F, DeKosky ST. Dopamine receptor genetic variation, psychosis, and aggression in Alzheimer disease. Arch Neurol. 1998;55:1335–1340. 9779662 10.1001/archneur.55.10.1335

[92] Holmes C, Smith H, Ganderton R, Arranz M, Collier D, Powell J, Psychosis and aggression in Alzheimer’s disease: the effect of dopamine receptor gene variation. J Neurol Neurosurg Psychiatry. 2001;71:777–779. 11723200 10.1136/jnnp.71.6.777 PMC1737623

[93] Erga AH, Dalen I, Ushakova A, Chung J, Tzoulis C, Tysnes OB, Dopaminergic and Opioid Pathways Associated with Impulse Control Disorders in Parkinson’s Disease. Front Neurol. 2018;9:109. 29541058 10.3389/fneur.2018.00109 PMC5835501

[94] Golden SA, Jin M, Heins C, Venniro M, Michaelides M, Shaham Y. Nucleus Accumbens Drd1-Expressing Neurons Control Aggression Self-Administration and Aggression Seeking in Mice. J Neurosci Off J Soc Neurosci. 2019;39:2482–2496. 10.1523/JNEUROSCI.2409-18.2019 PMC6435830 30655356

[95] Ende G, Cackowski S, Eijk JV, Sack M, Demirakca T, Kleindienst N, Impulsivity and Aggression in Female BPD and ADHD Patients: Association with ACC Glutamate and GABA Concentrations. Neuropsychopharmacology. 2016;41:410. 26040503 10.1038/npp.2015.153 PMC5130117

[96] Chaibi I, Bennis M, Ba-M’Hamed S. GABA-A receptor signaling in the anterior cingulate cortex modulates aggression and anxiety-related behaviors in socially isolated mice. Brain Res. 2021;1762:147440. 33745922 10.1016/j.brainres.2021.147440

[97] Agrawal J, Dwivedi Y. GABAA Receptor Subunit Transcriptional Regulation, Expression Organization, and Mediated Calmodulin Signaling in Prefrontal Cortex of Rats Showing Testosterone-Mediated Impulsive Behavior. Front Neurosci. 2020;14:600099. 33240041 10.3389/fnins.2020.600099 PMC7677587

[98] Ogawa S, Lubahn DB, Korach And KS, Pfaff DW. Aggressive Behaviors of Transgenic Estrogen-receptor Knockout Male Micea. Ann N Y Acad Sci. 1996;794:384–385. 8853623 10.1111/j.1749-6632.1996.tb32549.x

[99] Vaillancourt KL, Dinsdale NL, Hurd PL. Estrogen receptor 1 promoter polymorphism and digit ratio in men. Am J Hum Biol Off J Hum Biol Counc. 2012;24:682–689. 10.1002/ajhb.22297 22806965

[100] Horton BM, Hudson WH, Ortlund EA, Shirk S, Thomas JW, Young ER, Estrogen receptor α polymorphism in a species with alternative behavioral phenotypes. Proc Natl Acad Sci U S A. 2014;111:1443–1448. 24474771 10.1073/pnas.1317165111 PMC3910653

