# Supplementary figures and images for: Unique Transcriptional Signatures Correlate with Behavioral and Psychological Symptom Domains in Alzheimer’s Disease
Source: Res Sq. 2023 Jan 11:rs.3.rs-2444391. Preprint. [Version 1] doi: 10.21203/rs.3.rs-2444391/v1 (PMC9882691; doi:10.21203/rs.3.rs-2444391/v1)

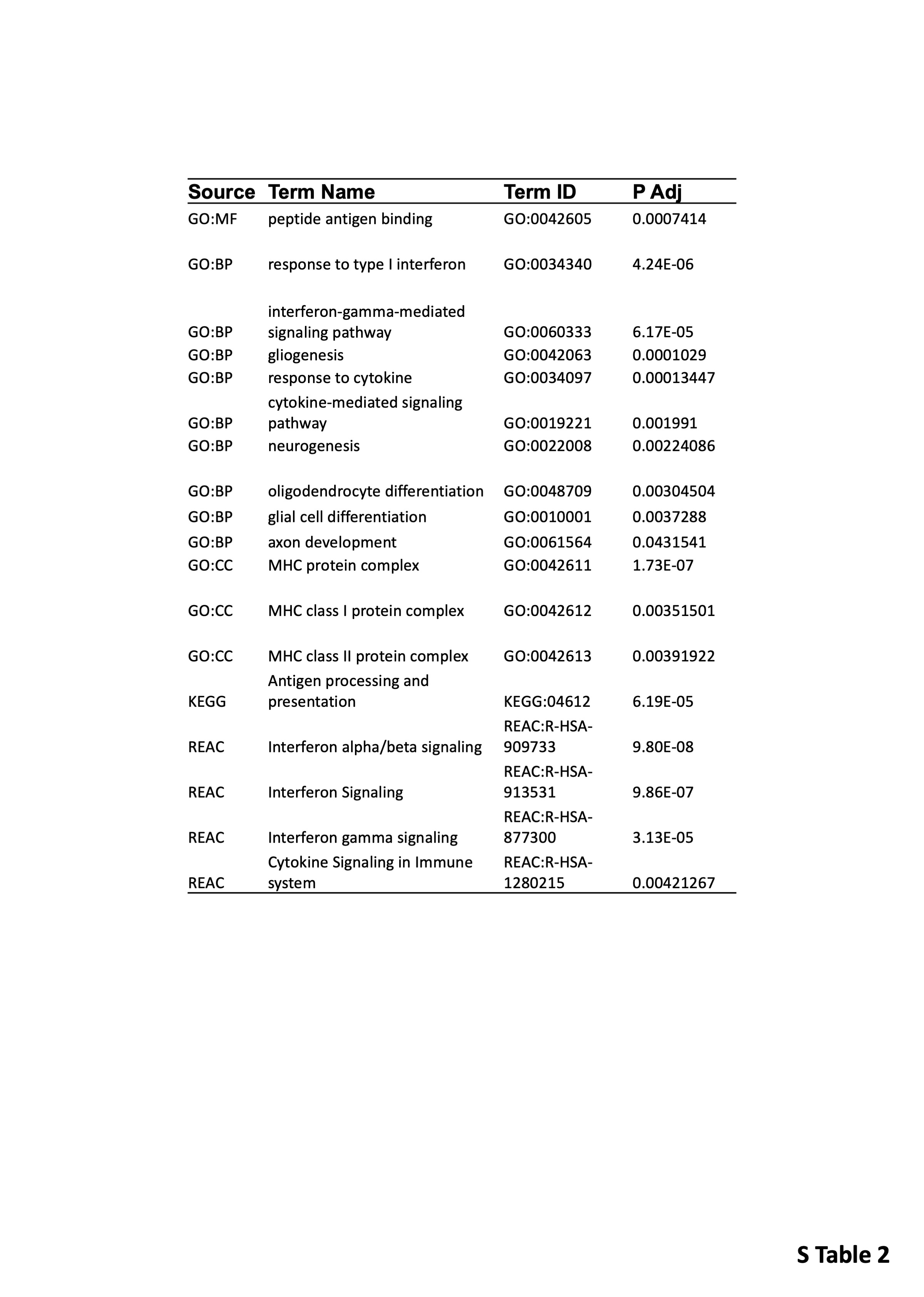

Supplement: Supplementary Table S2 — Notable Affective DEG Enrichments. Using gProfiler2, functional enrichment analysis was performed, and the most notable pathways are listed in this table. Source indicates the databases queried and include Gene ontology: Molecular function (GO:MF), GO: Biological process (GO:BP), GO: Cellular compartment (GO:CC), Kyoto encyclopedia of gene and genomes (KEGG), and Reactome (REAC). Adjusted p-values (P Adj) > 0.05 were considered significant. [file ressq-nihpprs2444391v1-r_suptable2.jpg]

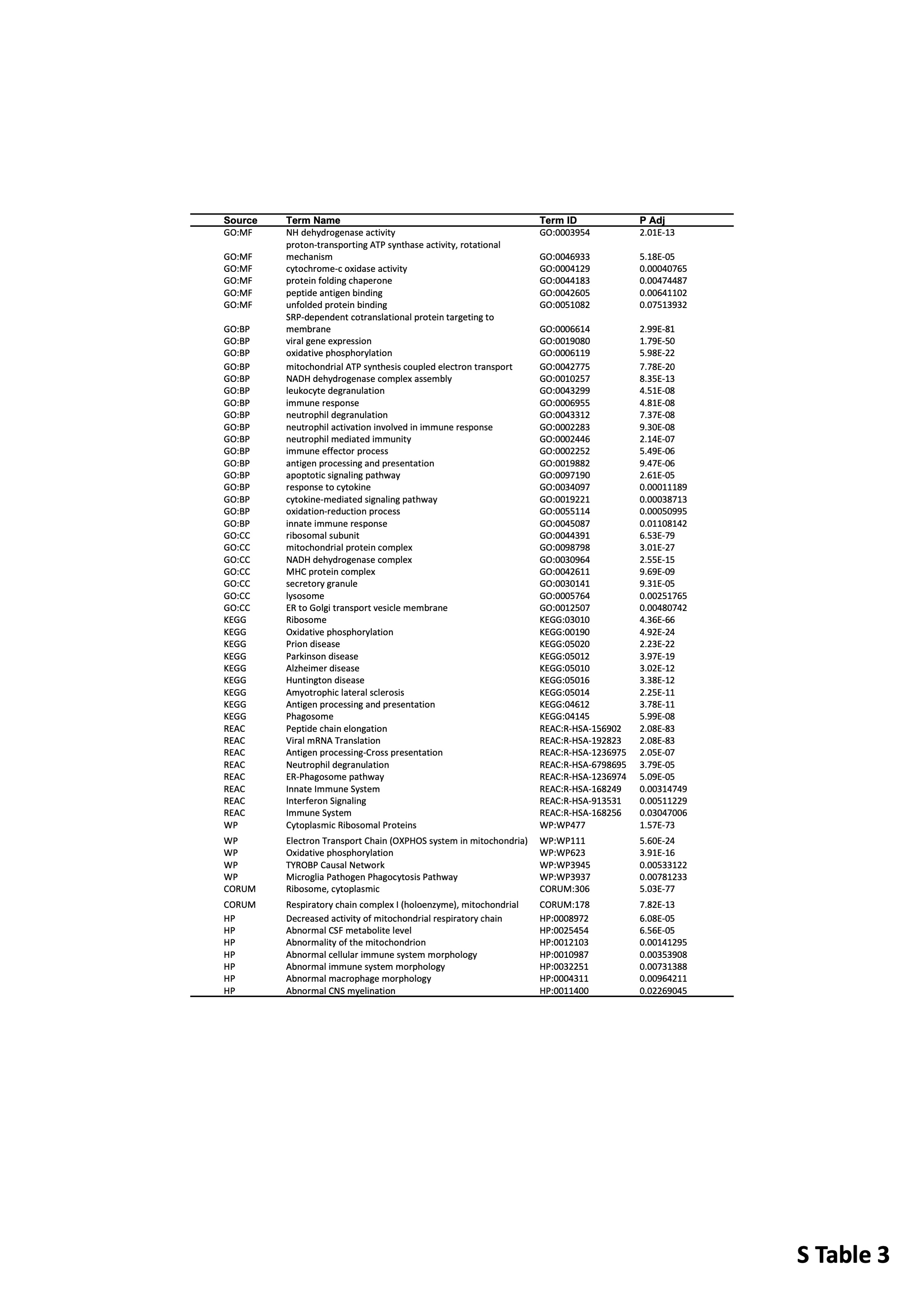

Supplement: Supplementary Table S3 — Notable Apathy DEG Enrichments. Using gProfiler2, functional enrichment analysis was performed, and the most notable pathways are listed in this table. Source indicates the databases queried and include Gene ontology: Molecular function (GO:MF), GO: Biological process (GO:BP), GO: Cellular compartment (GO:CC), Kyoto encyclopedia of gene and genomes (KEGG), Reactome (REAC), WikiPathways (WP), CORUM, and Human phenotype ontology (HP). Adjusted p-values (P Adj) > 0.05 were considered significant. [file ressq-nihpprs2444391v1-r_suptable3.jpg]

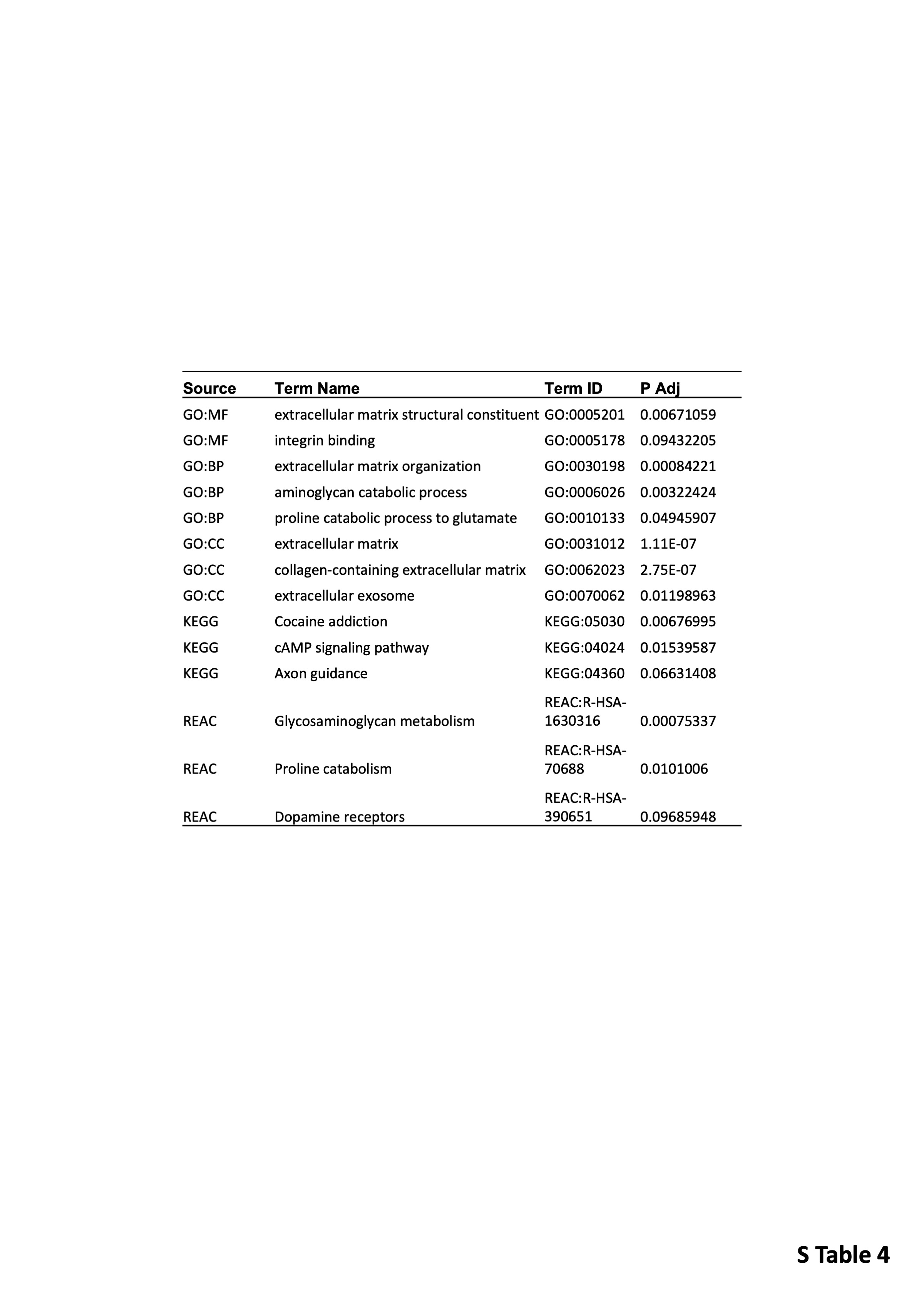

Supplement: Supplementary Table S4 — Notable Psychosis DEG Enrichments. Using gProfiler2, functional enrichment analysis was performed, and the most notable pathways are listed in this table. Source indicates the databases queried and include Gene ontology: Molecular function (GO:MF), GO: Biological process (GO:BP), GO: Cellular compartment (GO:CC), Kyoto encyclopedia of gene and genomes (KEGG), and Reactome (REAC). Adjusted p-values (P Adj) > 0.05 were considered significant. [file ressq-nihpprs2444391v1-r_suptable4.jpg]
