# Supplementary material for: Unique Transcriptional Signatures Correlate with Behavioral and Psychological Symptom Domains in Alzheimer’s Disease
Source: Res Sq. 2023 Jan 11:rs.3.rs-2444391. Preprint. [Version 1] doi: 10.21203/rs.3.rs-2444391/v1 (PMC9882691; doi:10.21203/rs.3.rs-2444391/v1)
Supplement: supplementary materials [file Supplementary_Materials_BPSD_and_AD_RNAseq.pdf]

Supplementary Materials

Supplementary Results and Discussion

Affective Domain

RESULTS

Though the affective domain had the fewest number of DEGs, there were still >600 DEGs by nominal p-value, and enrichment analysis yielded two broad categories of immune regulation (antigen processing and presentation, cytokine signaling, and interferon signaling) and cellular maturation (oligodendrocyte differentiation, glial cell differentiation, and neurogenesis; (Supplementary Table S2). It is notable that many type I and type II MHC genes were DEGs for this domain, including *HLA-A* ($M_{\text{Fold Change}}$ = 0.795, p = 0.045), *HLA-B* (M_{FC} = 0.771, p = 0.024), *HLA-C* (M_{FC} = 709, p = 0.006), and *HLA-E* (M_{FC} = 0.746, p = 0.023) as well as *HLA-DRB1* (M_{FC} = 0.718, p = 0.017), *HLA-DPA1* (M_{FC} = 0.737, p = 0.030), *HLA-DPB1* (M_{FC} = 0.761, p = 0.0094), *HLA-DMA* (M_{FC} = 0.787, p = 0.017), and *CD74* (*CLIP*, invariant chain of *MHC-II*; M_{FC} = 0.725, p = 0.036). In addition, the interferon pathways were highly enriched with the gamma-interferon receptor (IFNGR1; M_{FC} = 0.689, p = 0.014) being one of the DEGs. It was also notable that there was an enrichment for DEGs that are regulated by nuclear factor-kB (*NFKB*; Trans-Fac enrichment p_{adjusted} = 0.0069). These data may suggest alterations in the immune system, though potentially not restricted to innate immunity.

The affective domain was correlated with a growth factor and cell adhesion (greenyellow) module, also shared with agitation and psychosis, as well as a chaperone and cellular stress (grey60) module (Fig. 3A). The growth factor module was notably enriched for transforming growth factor-β (TGF-β), and similar to enrichment of all DEGs for the affective domain, genes tended to be regulated by *NFKB* (p_{adjusted} = 0.0008). In concordance, *TGFB1* (M_{FC} = 0.750, p = 0.003) was a notable hub gene as were *PLXNB1* (M_{FC} = 0.799, p = 0.023) and *INPPL1* M_{FC} = 0.756, p = 0.002), which were together reported as two important hub genes related to cognitive decline in AD and Aβ pathology in a large RNA-seq dataset from human brain tissue, and further validated as affecting Aβ-42 expression in cell culture[1]. The Chaperone module was a small transcriptional module with 38 total genes and was enriched primarily for chaperone response to misfolded proteins, especially genes related to HSP70 activity. Interestingly, two notable potential hub genes are immune system genes *CSF1* (M_{FC} = 0.809,

p = 0.037) and *NFKBIA* (M_{FC} = 0.810, p = 0.053), and this module was also enriched for genes regulated by *NFKB* ($a_{djusted}$ = 0.0089). Transcriptomic changes detected in affective cases did not coincide with differences in the abundance of any individual cell type (Supplementary Fig. S3A).

Divergent information flow was analyzed between cases and controls in the affective domain, and the top 40 network routers, key targets, and high impact genes are shown (Supplementary Fig. S4-5). Three high impact genes were especially notable. The first two were *TGFBR1*(Supplementary Fig. S 4F) and *TGFBRAP1* (Supplementary Fig. S 4G), which are one of the receptors and an auxiliary protein for *TGF-β*, respectively. The third high impact gene is *GSK3-β* (Supplementary Fig. S4H), which has a well-defined role in facilitating hyperphosphorylation of tau and Aβ production[2].

DISCUSSION

Overall, there are numerous potential pathways and genes that could be important for affective domain symptoms, but many of these center around the immune system. For many years now, numerous studies have hypothesized that the immune system plays a central role in AD pathogenesis, though there is likely a change in immune landscape that is more complex than it simply being increased or decreased, pro- or anti-inflammatory, or neurotoxic and neuroprotective[3, 4]. Interestingly, treatment-resistant depression has also been implicated as having an abnormal immune landscape, leading some to believe that defining certain major depressive disorders as immune driven could be an important distinction for understanding pathogenesis and treatment[5, 6]. As affective symptoms in AD are also often refractory to first-line treatment[7, 8], one may hypothesize affective symptoms in AD could have a similar mechanism. Notably, late-life depression, which could be a prodrome of AD in some cases[9], has some evidence to suggest a proinflammatory etiology[10].

However, most of the data on the immune system's role in depression involves peripheral immune markers, mostly cytokines like TNF-α and IL-6 or acute phase reactants like C-reactive protein (CRP)[11–16], whereas the majority of data for the immune system in AD pathogenesis involve central immune responses[4], though peripheral changes exist[17]. Though these peripheral responses may influence microglia, the exact link remains unclear. Therefore, it is interesting that so many of the HLAs, which are mostly associated with antigen

presentation and therefore adaptive immune responses, were DEGs for the affective domain. The role for adaptive immunity in classical, autoimmune neurodegenerative diseases like multiple sclerosis are well-established, but innate immunity has thought to be the greater driver of AD pathogenesis[4, 18]. Despite this, T-cells and B-cells, which are the main effector cells in adaptive immunity, are altered in the blood and CSF of AD patients in ways that suggest some cross-talk with central neurodegeneration[17–21]. In addition, two HLA loci were uncovered as GWAS hits associated with AD and frontotemporal dementia risk[22–24]. Different expression of MHC-II molecules have been reported in the brains of patients with AD compared to otherwise healthy controls[18, 25, 26]. Elevated T-cells and B-cells have also been reported in the CNS in AD, but they remain much diminished compared to other autoimmune neurodegenerative conditions[27, 28]. Finally, one study found elevated IL-6 and TNF-α in AD patients with depression compared to euthymic AD patients and sex matched controls[29], though it is not clear if this has been replicated. Another interesting point in this study is that cognition measured by MMSE correlated well with IL-6 and TNF-α in AD patients with depression but to a much lesser degree in AD patients without depression. It would be quite speculative to suggest an adaptive immune response driving affective symptoms in AD, though it is interesting to note that those with other autoimmune diseases tend to have mood disorders at a higher rate[6, 30, 31].

Targets of the transcription factor *NFKB* were consistently enriched in the DEGs and the two modules associated with the affective domain, with *NFKBIA* being a likely hub gene for the Chaperone module, which was unique to the affective domain. *NFKBIA* encodes an inhibitory subunit of the NFKB complex that prevents its translocation to the nucleus and further transcription of downstream targets. NFKB has a wide range of roles across numerous cell types, but it is central in proinflammatory responses involving cytokines, though again its role is likely more nuanced and depends on physiological context[32, 33]. However, *NFKB* also plays a role in neuronal function and survival when expressed in neurons[33], further complicating the role of *NFKB* in the affective domain. Still, in the context of the connection between *NFKB* and our data, it is notable that *NFKB* regulates, is regulated by, or has a bidirectional connection with many of the genes and pathways implicated in the affective domain. For instance, *NFKB* activation in microglia often lead to downregulation of TGF-β, which facilitates a transition to the damage-associated microglia (DAM) implicated in AD pathogenesis, though a

bidirectional relationship is likely[20, 33, 34]. NFKB is a downstream effector of interferon-gamma[35], and it is downstream of T-cell receptors when they interact with antigen presenting HLAs[36]. Interestingly, protein aggregation, such as misfolded tau, may even trigger downstream *NFKB* signaling, suggesting connection to the HSP70 proteins that also interact with misfolded proteins like p-tau[37–41]. Aβ may also lead to increased *NFKB* signaling in microglia[34, 42]. Due to these numerous associations, it is possible that imbalances in *NFKB* could promote affective symptoms in AD through multiple pathways and therefore may not represent a good therapeutic target but understanding more about how *NFKB* influences affective symptoms could be mechanistically important.

Finally, the association of *GSK3-β* with the affective domain may be of therapeutic significance, and it is interesting that this high impact gene was not implicated in the other domains. In addition, a prior purely bioinformatic study on protein-protein interactions we conducted implicated *GSK3-β* as being a major hub gene for the affective domain specifically[43]. As mentioned, *GSK3-β* is an important kinase that facilitates the hyperphosphorylation of tau and Aβ production[2], but it is also a key target for the drug lithium[44]. Similar to AD, depression in bipolar disorder is often refractory to SSRIs, but lithium is often a mainstay of both mania prevention and bipolar depression treatment[45]. Some small trials have also shown a pro-cognitive effect of lithium in AD, but further testing is needed to support this[46]. Healthcare providers may be hesitant to prescribe lithium to AD patients due to its narrow therapeutic window and need for close monitoring, but if its effect on inhibiting *GSK3-β* could lead to improvement in affective symptoms, it may be worth pursuing clinically at low doses. Further research into this area could be illuminating.

Apathy Domain

RESULTS

Of all the domains, the apathy domain had the highest percentage of unique DEGs based on a nominal p-value (776 genes, 80.8% of total DEGs), and most were downregulated – 825 out of 960 total DEGs. Similar to other domains, enrichment analysis of all the DEGs for the apathy domain was broad but ultimately centered on three processes: Ribosomes and translation, oxidative phosphorylation, and immune system regulation. The degree of

enrichment for ribosomal proteins was high, as 87.9% of the 40S subunits and 91.5% of the 60S subunits were DEGs, and all were downregulated (Supplementary Table S3). The DEGs for ribosomal proteins – which included small, large, and mitochondrial ribosomal proteins – are distinct from rRNA, which were removed as part of the pre-processing steps before RNA-seq, as is often standard. For oxidative phosphorylation genes, there were numerous subunits of the electron transport chain implicated including the NADH:ubiquinone oxidoreductase family (NDUF), cytochrome-c oxidase (COX), and ATP synthase proteins. While there was some overlap with the affective domain in terms of the immune system regulation pathways, it was notable that pathways related to innate immune activation, especially highlighting the microglial *TYROBP* causal network, were unique to the apathy domain. Some of these DEGs included *TREM2* (M_{FC} = 0.768, p = 0.021), *CD33* (M_{FC} = 0.766, p = 0.009), *TYROBP* (M_{FC} = 0.639, p = 0.002), *C1QA* (M_{FC} = 0.556, p = 5.5 $x10^{-5}$), *C1QB* (M_{FC} = 0.591, p = 4.1$x10^{-4}$), C1QC (M_{FC} = 0.589, p =1.4$x10^{-4}$), and *C3* (M_{FC} = 0.744, p = 0.005).

WGCNA revealed two networks of genes that significantly correlated with the apathy domain: the microglial phagocytic pathway and complement cascade module (Tan) was uniquely correlated (r = -0.38, p = 0.003), whereas the ECM module (Dark Turquoise) significantly correlated with the apathy domain (r = -0.30, p = 0.020) in addition to the psychosis and agitation domains (Fig. 3A). The Microglia module was enriched for both innate and adaptive immune responses, though innate immune processes often had more significant enrichments. In addition, microglial phagocytosis, complement activation, and the TYROBP Causal Network were enriched for this module. Within the 77-gene Microglia Module, 43 genes met the WGCNA criteria for candidate hub gene designation (Supplementary Table S2), including *C1QA, C1QB, C1QC, C3,* and *TYROBP*. Analysis of cell type abundance indicated significantly fewer microglia in apathy cases compared to controls, *p* = 0.008 (Supplementary Fig. S2B).

Divergent information flow was analyzed between cases and controls in the apathy domain, and the top 40 network routers, key targets, and high impact genes are shown (Supplementary Fig. S6-7). Three high impact genes were notable: *TRAF6* (Supplementary Fig. S6F) and *CUL1* (Supplementary Fig. S6G) and, which are components of E3 ubiquitin ligase complexes, and *CREBBP* (Supplementary Fig. S6H), a lysine acetyltransferase.

DISCUSSION

The most striking difference between the transcriptomes of cases with apathy compared with controls was the overabundance of ribosomal proteins as DEGs followed by a similarly large number of Electron Transport Chain (ETC) components. While the role of mitochondrial dysfunction and subsequent metabolism is well studied in AD and may dovetail with accelerated aging[47–49], the role of ribosome biogenesis and maintenance may not be as well studied. The rate of protein synthesis is generally considered to be reduced in AD[50] and AD mouse models[51], but whether ribosomal proteins are downregulated in AD is less clear, as some groups have found decreases in rRNA but not polyribosomes[50, 52] while others have found decreases in specific ribosomal proteins that are more prominent with advancing pathological stage[53]. The local subcellular maintenance of ribosomal proteins for dendrites and axons is essential for the continued function of these structures[54, 55], and disinhibition of S6-mediated translation leads to cognitive benefits in an AD mouse model[56]. On the translational level, hyperphosphorylated tau has been shown to stall translation by direct interaction with the ribosome, potentially leading to an inability to maintain neuronal proteins[57]. It is possible that apathy becomes more likely to develop in those with depleted ability to maintain ribosomal proteins via reduced transcription of these subunits when compared to other AD patients. Interestingly, protein synthesis also decreases with age, though it is unclear whether ribosomal proteins go up, down, or are more finely regulated at the individual subunit level [58–61]. Whether the effects of poor aging could also intersect with increased apathy is a question that deserves more investigation.

While the immune system was associated with both the affective and apathy domains, pathways related to innate microglial functions were uniquely represented by the DEGs associated with apathy. Similar to how apathy and depression can be difficult to parse clinically but are clearly separable[62, 63], the differentiating features in immune function between these domains may be subtle but important in terms of mechanism. In particular, there was a unique link between the apathy domain and microglial proteins already implicated in AD pathogenesis, such as *TREM2*[64], *CD33*[65], *TYROBP*[66, 67], and the complement cascade[68, 69]. Despite this clear link, the apathy domain was also associated with decreased estimated microglia overall, which makes

interpreting the decrease in immune system genes more challenging. It may also seem counterintuitive that those with more severe neuropsychiatric symptoms like apathy would have fewer microglia and potentially neuroinflammation. However, these findings may indicate that the participants with apathy in our cohort may have been at more advanced stages of AD before death, as the number of patients with apathy often increases with disease severity[70, 71]. Similarly, there is a reduction in microglia associated with the most developed amyloid plaques and the highest Braak stage[72, 73]. Combined with the DEG enrichment of dysregulation of ribosomal protein expression and energy production via oxidative phosphorylation, the increase in apathy in this cohort could be more representative of the most severely progressed AD and be distinct from apathy in earlier stages. However, other hypotheses should also be considered.

Going beyond the potential that lowered microglia and their associated genes are simply a result of end-stage disease, akin to 'burnout', it is intriguing to consider that a sub-optimal microglial response may lead to apathy. For instance, while AD is associated with higher levels of *TYROBP* when compared to cognitively unimpaired controls[67], our analysis demonstrated that among AD patients, those with apathy may have lower expression of this gene. Therefore, it may be that those with apathy are unable to mount as strong an immune response as those without, despite both sets of AD patients having higher microglial responses compared to those that are not afflicted. Many of the downregulated immune genes are necessary for efficient microglial efferocytosis, which is thought to be neuroprotective, and so a reduced ability to engage in this process could lead to the underlying cellular dysfunction in AD patients with more prominent apathy[74]. Preliminary results from a separate experiment in our laboratory suggest that *TYROBP* mRNA expression in the ACC is indeed higher in cases of apathy in AD compared to controls without dementia but lower than those with AD and no apathy (data not shown). Whether this pattern of intermediate expression in AD apathy cases compared to non-apathetic AD and cognitively unimpaired individuals is pervasive among the rest of the Microglial module genes remains to be seen, and it is also unclear if a very specific pattern of reduced phagocytosis and complement activation amongst microglia is the main driver.

Though ribosomal biogenesis and maintenance were implicated through the downregulation of ribosomal proteins, two notable regulators of proteostasis were implicated in the network flow analysis, TRAF6 and

CUL1. TRAF6 is an ubiquitin E3 ligase that is best known for being downstream of the TNF-α and activating *NFKB*[75] while CUL1 forms an ubiquitin E3 ligase complex with SKP1, RBX1 or RBX2, and F-box proteins (forming a complex known as SKP1-CUL1-F-Box, SCF) and coordinates the degradation of various proteins, ultimately affecting numerous cellular functions[76]. The E3 ligases facilitate the ubiquitination of many proteins, which affects the degradation or aggregation of these proteins, including those involved in neurodegeneration[77]. Specifically, TRAF6 and SCF have been shown to ubiquitinate polyQ proteins like Huntingtin[78–80] and α-synuclein[81, 82]. In AD, these E3 ligases are also likely to contribute to pathogenesis. CUL1 has been found to be downregulated in multiple brain regions in AD[83] and affects APP processing via its ubiquitination of presenilin[84]. TRAF6 is generally upregulated in AD brains[85–87], ubiquitinates gamma-secretase[88] and tau[89], is inhibited from promoting cell survival by Aβ via lack of polyubiquitination of p75(NTR)[90], and also is downstream of Aβ-induced activation of the NFKB pathway[87]. While the cross-talk between these E3 ligases and ribosomal proteins is relatively unexplored, the pairing of these two processes could suggest that overall protein regulation is aberrant in those with AD and apathy. Interestingly, E3 ligases have also been tied to regulating AMPA and NMDA receptors[91, 92], and it is possible that overall aberrant regulation of postsynaptic proteins through multiple mechanisms is involved in apathy in AD.

Network flow analysis also suggested that *CREBBP* may point of increased information flow in the system. *CREBBP*, often further abbreviated as CBP, shares significant homology with p300, and both enzymes act as lysine acetyltransferases, including of histones Both proteins have significant overlapping functions and bind to CREB, which makes determining differences in substrate specificity between two enzymes challenging[93]. CBP/p300 have both been implicated in AD, but the overall mechanism of influence is complex. For instance, CBP/p300 was found to be reduced in the frontal cortex and trended towards reduction in the hippocampus of human AD patients[94] and is decreased by Aβ via N-cadherin CTF without altering CBP transcription[95]. In mouse models of AD including those driven by Aβ and mutant tau, interventions to increase CBP/p300 activity result in improved memory performance[96, 97]. In addition, increased CBP/p300 activity has been found to lead to acetylation of histones at both PS1 and BACE1 in N2a cells with the APP-Swedish mutation, suggesting

that increased CBP/p300 could lead to more pathogenic Aβ[98]. These lines of evidence would suggest that decreasing CBP/p300 leads to worsening AD and that CBP/p300 potentiators could be a via therapeutic target. However, other groups have found the CBP/p300 activity at various histone markers is increased in the CSF and brains of AD patients[99, 100] and may have increased activity induced by Aβ via S-nitrosylated GAPDH[101]. Interestingly, CBP/p300 also directly acetylates tau, and this leads to maintenance of aggregation-prone p-tau[102] as well as aberrant translation and reduced expression of rRNA and ATP[103]. These findings are even more intriguing in light of a recent study that found that Traumatic Brain Injury (TBI) increases Tau acetylation via CBP/p300, again via S-nitrosylated GAPDH, and leads to aberrant tau localization[104]. Blocking the acetylation of tau in a mouse model of AD led to decreased neurodegeneration and improved memory behaviors. It should also be noted that those taking a CBP inhibitor, salsalate, had a reduced risk of developing both TBI and AD after a 6 year follow-up[104]. Similar to AD, TBI is a neurological disease that is known to have high rates of apathy[105], and one could speculate that these changes in acetylated tau could lead more significantly to apathy in affected patients.

Psychosis Domain

RESULTS

The overlap of DEGs with the agitation domain led to considerable overlap in the enrichment analysis, especially for pathways related to the ECM (Supplementary Table S4). However, a few distinct pathways are enriched for psychosis domain DEGs and include cocaine addiction, cAMP signaling, and dopamine receptors, mainly *DRD1* (M_{FC} = 1.38, p = 0.006), *DRD2* (M_{FC} = 1.42, p = 0.012), and *DRD4* (M_{FC} = 0.744, p = 0.022). In addition, all 3 genes involved in the catabolic process to turn proline into glutamate were DEGs, *PRODH* (M_{FC} = 0.744, p = 0.017), *PRODH2* (M_{FC} = 0.727, p = 0.004), and *ALDH4A1* (M_{FC} = 0.799, p = 0.023).

The psychosis domain was associated with three shared modules and three unique modules. For the shared modules, while a weaker correlation was seen for the chaperone (greenyellow) module, overlapping with the affective and agitation domains, the strongest correlations were seen for the ECM (darkturquoise) and transcription factor (magenta) modules, overlapping with the agitation domain. While the connections between

the agitation domain and ECM are discussed in the main text, the transcription factor module was enriched for many transcription factors, with *PLAGL1* (M_{FC} = 0.447, p = 2.3 x 10^{-6}) and *RUNX3* (M_{FC} = 0.666, p = 1.0 x^{-5}) being important hub genes. *CD33* (M_{FC} = 0.699, p = $2.6x10^{-4}$) was another potential hub gene for this module, despite not being a transcription factor. For the three unique modules associated with the psychosis domain, there was a 77-gene astrocyte module (lightcyan) and two modules of unknown function (cyan and orange). The Astrocyte module was enriched with pathways related to astrocyte function and astrocytic projections (Fig. 3A). Hub genes include astrocyte-related proteins *MLC1* (M_{FC} = 0.780, p = 0.019), *SNTA1* (M_{FC} = 0.830, p = 0.032), and *PLTP* (M_{FC} = 0.764, p = 0.002) as well as *APOE*, which was not a significant DEG. The cyan and orange modules were relatively small, with 58 and 20 genes, respectively, and enrichment analysis did not suggest a specific function. Notable hub genes for these modules include *DVL1* (M_{FC} = 0.864, p = 0.071)and *CDKN1C* (M_{FC} = 0.578, p = 0.007) for the cyan module and *INO80D* (M_{FC} = 0.726, p = 0.021) and *CEACAM21* (M_{FC} = 0.684, p = 0.185) for the orange modules. Transcriptomic changes detected in psychosis cases did not coincide with differences in the abundance of any individual cell type (Supplementary Fig. S2C).

Divergent information flow was analyzed between cases and controls in the psychosis domain, and the top 40 top 40 network routers, key targets, and high impact genes are shown (Supplementary Fig. 8-10). While DEGs between the agitation and psychosis domain showed pronounced overlap, it was surprising that much less overlap was seen in terms of the top 40 impact genes in this analysis, with only 25% shared between the domains. Four high impact genes were notable: *HSP90AA1, ARRB1, YWHAZ*, and *NR3C1*. *HSP90AA1* is one of the primary subunits of *HSP90*, which has been implicated in AD based on its role in handling misfolded proteins, especially tau[106–108]. AARB1 is a β-arrestin that is downstream of GPCRs like the dopamine receptors and shunts GPCRs into non-cAMP secondary messaging[109–111]. YWHAZ is a phosphoserine binding protein of the 14-3-3 family that is bound to tau in neurofibrillary tangles[112]. NR3C1 is the glucocorticoid receptor, and glucocorticoid signaling has been shown to be altered in AD[113]. Finally, one key target and one network router were notable and unique to this domain, *HTT* and *SCNA*, respectively. *HTT* encodes huntingtin, which is the pathogenic protein in Huntington's disease[114], and information flow in this small network switched from DLG4 (PSD-95), which is a main post-synaptic scaffolding protein, to multiple

chaperones involved in the misfolded protein response. SCNA encodes α-synuclein, which is the pathological hallmark protein in Lewy body disorder, including Lewy body dementia and Parkinson's disease dementia[115]. Both Huntington's disease and Lewy body disorders have psychosis as prominent symptoms along with cognitive decline[116].

DISCUSSION

Disambiguating the differences between agitation and psychosis in this analysis are challenging given the overlapping design, but the shared transcriptional differences implicating ECM pathology are of interest. Despite the caveat that psychosis in primary psychiatric disorders may differ genetically from psychosis in AD, it is notable MMP-9 is elevated in the blood of those with schizophrenia[117]. The ECM module contained three genes related to the actin cytoskeleton as potential hub genes, *TPM2*, *TAGLN*, and *ACTA2*. It was surprising to find that a study examining induced pluripotent neural stem cells from bipolar disorder patients with history of psychosis had these 3 genes out of 42 total DEGs identified between patients and healthy controls[118]. These links between the ECM and psychosis are limited but may be interesting to investigate further.

In addition, the same paper that implicated *TAGLN*, *TPM2*, and *ACTA2* with bipolar disorder and psychosis noted *CEACAM21*, a potential hub gene in the orange module, as another one of the 42 DEGs[118]. Though *CEACAM21* is not a ECM protein, it was implicated in a Jewish-Israeli population GWAS as contributing to schizophrenia risk with validation of this allele in an independent Arab-Israeli cohort[119]. As this GWAS was done on an ethnically distinct population compared to the previous larger scale GWAS of schizophrenia, it is possible that there is a difference in polygenic risk and that this population aligns more closely with the genetics underlying AD with psychosis. This, however, is highly speculative.

Given that antipsychotics are first-line in treatment of schizophrenia and very commonly used for manic episodes with psychosis, perhaps it wasn't surprising that three of the five dopamine receptors, DRD1, DRD2, and DRD4, were DEGs. Despite this, evidence suggests that genetic data in schizophrenia is not enriched for the dopamine system[120]. As mentioned before, psychosis in AD may be distinct in terms of mechanism from

psychosis in primary psychiatric disorders, and the efficacy of antipsychotics in psychosis in dementia is unclear[121]. However, it is interesting to note that typical antipsychotics, which have a higher affinity for the dopamine 2 receptor (D2R), had better evidence for treatment of psychosis than atypical antipsychotics, which depend more on the combination of D2R and serotoninergic receptor blockage[122]. A challenge for using dopaminergic antagonists and partial agonists in AD with psychosis is the narrow therapeutic window, which may be a function of aging-related pharmacodynamic changes secondary to reduced expression of these receptors due to epigenetic alterations[123, 124].

Despite the FDA's black box warning for increased mortality with antipsychotic use in dementia[125], it is clear these medications and similar strategies manipulating dopaminergic or serotonergic tone are going to be used clinically for the foreseeable future, and so further modulation of the downstream signaling pathways may be important for drug development[123, 124]. Therefore, it was of great interest that our analysis uncovered evidence of changes in second messenger signaling pathways, mainly cAMP and β-arrestin. Most monoamines, including dopamine, serotonin, noradrenalin, and glutamate, target GPCRs as part or all of their mechanisms of action[126]. Though GPCR signaling is incredibly complex, the canonical pathways for G_{α} and G_{i} subunits affect cAMP second messenger signaling[127]. Often with repeated activation of the same GPCR, these G-coupled pathways get replaced by ones involving β-arrestins, which can lead to internalization of the GPCR as part of desensitization as well as an emerging role as downstream signaling molecules in their own right[128]. In our analysis, functional enrichment suggested that multiple genes involved in cAMP signaling were altered while our context-dependent analysis suggested that β-arrestin1 was the top key target and among the top high impact genes.

The two β-arrestins, 1 and 2, are expressed in the brain, though the expression of ARRB1 is almost 10 times higher in most brain regions than ARRB2[128]. However, both arrestins have been shown to have roles in facilitating the increase of the putative pathogenic drivers of AD, Aβ[128, 129] and p-tau[130, 131]. While both arrestins have been shown to be increased in frontotemporal dementia[130, 131], their changes in expression in AD is less clear[129]. The β-arrestins have nearly 78% homology[131], and knockout studies support some compensation of the roles of one receptor for the other, though different affinities for individual GPCRs and

downstream signaling are noted[129, 131, 132]. Both β-arrestins can play a role in dopaminergic signaling and regulate internalization of the receptor[109–111], further suggesting that signaling through these GPCRs and others could facilitate psychosis in AD.

In contrast to ARRB1 showing increased information flow in cases versus controls, YWHAZ showed the opposite association. YWHAZ is a 14-3-3 protein, which is a prominent scaffolding protein that facilitates protein-protein interactions and makes up nearly 1% of the brain's total proteins[133]. 14-3-3 has a wide-variety of functions that have been implicated in neurodegeneration including axon destabilization, p-tau-mediated pathogenicity, promoting formation of aggresomes and facilitating chaperone responses to misfolded proteins, and facilitating changes in synaptic plasticity[133–136]. However, its role as facilitating protein trafficking through endosomes, including recycling GPCRs to the cell surface, is particularly notable, involving the β-arrestins[137] and has been demonstrated for D2R[138]. Soluble 14-3-3 proteins are reduced in the temporal cortex in AD and Lewy body dementia[139], and it is notable that YWHAZ is the most common 14-3-3 protein that is found in neurofibrillary tangles in AD[112, 133, 140] and has been found as an effector of tau phosphorylation[133, 141]. In addition, genetic deletion of *YWHAZ* in mice leads to changes in dopamine-dependent behaviors that are ameliorated with D2R blocking antipsychotics[142]. One group has suggested that 14-3-3 proteins could be important for post-internalization sorting of GPCRs after association with the β-arrestins, with 14-3-3 helping to facilitate outcomes such as returning to the cell membrane or proceeding for degradation in lysosomes[137, 138]. Combined with the changes in dopamine receptor expression, cAMP signaling, ARRB1, and YWHAZ, one could hypothesize that changes in dopamine signaling, both on the level of receptor trafficking and downstream signaling, could serve as one mechanism leading to psychosis in AD.

Similar to *ARRB1, HSP90AA1* was found to be the top network router and among the top high impact genes showing increased information flow between cases and controls. This gene encodes the stress-inducible form of the HSP90 protein[143], and in contrast to the function of a similar chaperone, HSP70, HSP90 may maintain oligomeric p-tau, leading to worsening of AD pathogenesis[106, 107]. In addition, tau-driven changes in chromatin in AD may be reversible with HSP90 inhibitors[108]. Whether an enhanced role for p-tau's toxicity leads to a higher likelihood of psychosis in AD is unknown, though it is interesting that many studies report

increased neurofibrillary tangles in neocortical structures in AD with psychosis than in AD without psychosis as well as increased p-tau using a PET ligand[144, 145].

The final association worth mentioning is that all three genes involved in proline's metabolism to glutamate were among the DEGs for the psychosis domain. Because two of these genes fall within the 22q11.2 locus implicated in DiGeorge Syndrome, a disease causing neurodevelopmental dysfunction and psychosis, the role of proline in psychosis in primary psychiatric disorders has gained some attention[146]. Specifically, mutations in PRODH (proline dehydrogenase) or ALDH4A1 (Delta-1-Pyrroline-5-Carboxylate Dehydrogenase) can cause type I and type II hyperprolinemia, respectively, and these disorders often lead to psychosis, even when a full 22q11.2 deletion is not present[147]. Some studies have further found an interaction for increased psychosis between PRODH and another 22q11.2 gene, COMT, which metabolizes catecholamines like dopamine[147]. Some case-control studies have suggested hyperprolinemia in schizophrenia[147]. One study has also found a slight increase in proline in the plasma of AD or MCI patients[148] and another an association with Aβ+ and reduced cognition[149]. If and how elevated proline levels affect psychosis is unclear and could be related to or a proxy for abnormal glutamate metabolism, but there is some evidence that proline itself could alter GABAergic signaling[150]. Regardless, if abnormal proline turnover could be related to AD with psychosis, it may lend itself to be investigated by relatively non-invasive assays, such as urine screens for proline metabolites, which could be cheap tests to predict psychosis risk in AD.

SUPPLEMENTARY METHODS

Functional Enrichment Analysis

Functional enrichment analysis was performed using gProfiler2[151], and we included databases Gene Ontology (GO) – molecular function, GO – cellular localization, GO – biological function, Kyoto encyclopedia for gene G (KEGG), Reactome (REAC), TransFac (TF), Human protein atlas (HPA), Human phenotype ontology (HP), and CORUM. gProfiler2 identifies enriched genes using a hypergeometric test to demonstrate overrepresentation of certain genes among annotated pathways. gProfiler2 uses a 'Set Counts and Sizes' correction for multiple comparisons, which "considers the dependency of multiple tests by taking into account

the overlap of functional terms.” This yields an adjusted p-value that is more conservative than Benjamini-Hochberg false discovery rate but not as strict as Bonferroni correction. We determined an enriched pathways to have a p-adj <0.05, though we included pathways with $p < 0.1$ in our supplementary data. Functional Enrichment Analysis was performed on all DEGs and in the genes from each module.

WGCNA

Weighted gene co-expression network analysis (WGCNA) was performed to identify modules of gene co-expression[152, 153]. We included only the top 20% most variable genes by overall expression. We used a ‘signed’ analysis, which requires beta values twice as high as an ‘unsigned’ analysis. While soft-thresholding can be used to determine beta, there is little guidance about the biological relevance of changing three other parameters that change module association: DeepSplit, Merge CutHeight, and Minimum Module Size. To validate which values would yield the best biological significance of the resultant modules, we performed a WGCNA optimization analysis using an approach developed by Abbassi-Daloii[154]. Hypothesizing that *a priori* knowledge of the pattern of gene interactions could be used to create more biologically informed decisions about WGCNA parameters, especially when analyzing two highly similar tissue types, this approach incorporates data on known biological process from annotated databases to determine if resultant WGCNA modules are more or less biologically relevant. Specifically, the algorithm repeats the core WGCNA function multiple times for different permutations of the above mentioned variables. Then, it identifies co-expressed pairs of genes in each module and genes that are not co-expressed in the same module. Using an annotated database, and in this analysis using Reactome, all gene pairs are inspected for their present in the same biological pathways, and are counted as either having known biological connections or not. The four resultant groupings from this process are then analyzed with a Fisher’s Exact Test, and an odds ratio called an enrichment factor (EF) is created. A higher EF denotes more overlap with co-expressed pairs in the dataset with genes that are known to be part of the same biological process. Results of this pre-WGCNA analysis are included in (Supplementary Fig. S11).

A post-graduate biologist inspected the modules yielded by all combinations of parameters that with an EF > 1.5 and sought to balance a minimum grey module size (uncorrelated genes), high number of annotated genes included, relatively large number of modules, and biological relevance of resultant modules. Out of 420 permutations, the 406th yielded the best balance of these factors and also had incidentally had the highest EF. Accordingly, WGCNA was performed with the following parameters: beta = 22 (signed), DeepSplit = 4, Minimum Module Size = 20, Merge CutHeight 0.25.

This resulted in 15 modules with 1 uncorrelated 'grey' module. For each module, a potential hub gene was defined similar to previous guidance by WGCNA creators as having a module membership (MM) >0.8 and gene significance (GS) of >0.2. While all potential hub genes are available in our supplementary data, notable hub genes in Figure 2 were identified based on considerations of their high MM, high GS, overall high fold change in expression between cases and controls, and significant presence in the literature as affecting either behaviors in a particular domain or relevance to AD pathogenesis.

NetDecoder

While WGCNA gives an unbiased estimate of transcriptional modules based on correlations between expressed genes, biological context for a given change in expression is important in determining the diverse phenotypes that emerge. Interactions between proteins affect and are affected by transcriptional differences between cases and controls, and specific binding patterns, regulation by post-translational modulation, and other interactions that affect whether a protein is active or inactive can be modeled in terms of 'information flow' within a cellular network. NetDecoder[155] aims to analyze the context-dependent differences in cellular networks, such as DEGs in cases and controls, and determine how differences in this information flow differentiate two different cellular states. This algorithm relies on the well-conserved structure of protein-protein interactions and a "process guided flow algorithm to identify molecular interaction paths that connect a source gene (where information flow begins) to a target gene (also called sink, where information flow ends) with shared biological processes ." In doing so, the algorithm defines three types of 'key intermediary genes' in the network: 1) Network Routes, defined as intermediary proteins that influence many genes and have high difference in

information flow when comparing two networks; 2) Key targets, defined as sink nodes that have the highest differences in flow between the two compared networks; 3) High impact genes, defined as genes that experience a significant change in regulation between the two compared networks, which include flow difference, establishment of new inflows, and change in directionality of in gene expression between the two phenotypes. By definition, a high impact gene can be a network router or key target, but a network router and key target are mutually exclusive. To determine which genes are high impact genes, a novel scoring system created an impact score that was the product of the three aforementioned parameters that define a high impact gene.

In our analysis, we used the DEGs in each domain as the source gene, while sink genes remained defined as those that directly regulate transcription. Because DEGs were used as source genes, and because WGCNA uses Gene Significance, a measure that heavily relies on differential expression, to identify potential hub genes, we did not expect overlap in genes highlighted by NetDecoder and WGCNA's potential hub genes. For our interaction network, we used the previously constructed R object from the NetDecoder publication, which was created as follows: via iRefIndex v14.0, an interaction network was constructed using all available interactions while removing self-loops and multiple edges. We completed 4 separate NetDecoder analyses, one using the DEGs that were specific to each behavioral domain. We kept the top 20 positive and top 20 negative genes in terms of flow difference or impact score for visualization across the three intermediary genes. For visualizing changes in overall domain networks or subnetworks relating to the intermediary genes, we used Cytoscape. To simplify visualization, we filtered out edges where there was very little difference in flow, thus highlighting the results with the largest effects on the networks.

REFERENCES

1. Mostafavi S, Gaiteri C, Sullivan SE, White CC, Tasaki S, Xu J, et al. A molecular network of the aging human brain provides insights into the pathology and cognitive decline of Alzheimer's disease. Nat Neurosci. 2018;21:811–819.
2. Hernandez F, Lucas JJ, Avila J. GSK3 and Tau: Two Convergence Points in Alzheimer's Disease. J Alzheimers Dis. 2013;33:S141–S144.

3. Sarlus H, Heneka MT. Microglia in Alzheimer's disease. J Clin Invest;127:3240–3249.
4. Heppner FL, Ransohoff RM, Becher B. Immune attack: the role of inflammation in Alzheimer disease. Nat Rev Neurosci. 2015;16:358–372.
5. Beckett CW, Niklison-Chirou MV. The role of immunomodulators in treatment-resistant depression: case studies. Cell Death Discov. 2022;8:367.
6. Beurel E, Toups M, Nemeroff CB. The Bidirectional Relationship of Depression and Inflammation: Double Trouble. Neuron. 2020;107:234–256.
7. Banerjee S, Hellier J, Dewey M, Romeo R, Ballard C, Baldwin R, et al. Sertraline or mirtazapine for depression in dementia (HTA-SADD): a randomised, multicentre, double-blind, placebo-controlled trial. The Lancet. 2011;378:403–411.
8. Dudas R, Malouf R, McCleery J, Dening T. Antidepressants for treating depression in dementia. Cochrane Database Syst Rev. 2018;2018:CD003944.
9. Heser K, Bleckwenn M, Wiese B, Mamone S, Riedel-Heller SG, Stein J, et al. Late-Life Depressive Symptoms and Lifetime History of Major Depression: Cognitive Deficits are Largely Due to Incipient Dementia rather than Depression. J Alzheimers Dis JAD. 2016;54:185–199.
10. Alexopoulos GS. Mechanisms and treatment of late-life depression. Transl Psychiatry. 2019;9:188.
11. Chamberlain SR, Cavanagh J, de Boer P, Mondelli V, Jones DNC, Drevets WC, et al. Treatment-resistant depression and peripheral C-reactive protein. Br J Psychiatry J Ment Sci. 2019;214:11–19.
12. Strawbridge R, Arnone D, Danese A, Papadopoulos A, Herane Vives A, Cleare AJ. Inflammation and clinical response to treatment in depression: A meta-analysis. Eur Neuropsychopharmacol J Eur Coll Neuropsychopharmacol. 2015;25:1532–1543.
13. Strawbridge R, Hodsoll J, Powell TR, Hotopf M, Hatch SL, Breen G, et al. Inflammatory profiles of severe treatment-resistant depression. J Affect Disord. 2019;246:42–51.
14. Cattaneo A, Gennarelli M, Uher R, Breen G, Farmer A, Aitchison KJ, et al. Candidate Genes Expression Profile Associated with Antidepressants Response in the GENDEP Study: Differentiating between Baseline 'Predictors' and Longitudinal 'Targets'. Neuropsychopharmacology. 2013;38:377–385.
15. Lauden A, Geishin A, Merzon E, Korobeinikov A, Green I, Golan-Cohen A, et al. Higher rates of allergies, autoimmune diseases and low-grade inflammation markers in treatment-resistant major depression. Brain Behav Immun - Health. 2021;16:100313.
16. Kiraly DD, Horn SR, Van Dam NT, Costi S, Schwartz J, Kim-Schulze S, et al. Altered peripheral immune profiles in treatment-resistant depression: response to ketamine and prediction of treatment outcome. Transl Psychiatry. 2017;7:e1065.
17. Bettcher BM, Tansey MG, Dorothée G, Heneka MT. Peripheral and central immune system crosstalk in Alzheimer disease — a research prospectus. Nat Rev Neurol. 2021;17:689–701.
18. Aliseychik MP, Andreeva TV, Rogaev EI. Immunogenetic Factors of Neurodegenerative Diseases: The Role of HLA Class II. Biochem Mosc. 2018;83:1104–1116.
19. Gate D, Saligrama N, Leventhal O, Yang AC, Unger MS, Middeldorp J, et al. Clonally expanded CD8 T cells patrol the cerebrospinal fluid in Alzheimer's disease. Nature. 2020;577:399–404.
20. Laurent C, Buée L, Blum D. Tau and neuroinflammation: What impact for Alzheimer's Disease and Tauopathies? Biomed J. 2018;41:21–33.
21. Kim K, Wang X, Ragonnaud E, Bodogai M, Illouz T, DeLuca M, et al. Therapeutic B-cell depletion reverses progression of Alzheimer's disease. Nat Commun. 2021;12:2185.
22. Lambert J-C, Ibrahim-Verbaas CA, Harold D, Naj AC, Sims R, Bellenguez C, et al. Meta-analysis of 74,046 individuals identifies 11 new susceptibility loci for Alzheimer's disease. Nat Genet. 2013;45:1452–1458.
23. Ferrari R, Hernandez DG, Nalls MA, Rohrer JD, Ramasamy A, Kwok JBJ, et al. Frontotemporal dementia and its subtypes: a genome-wide association study. Lancet Neurol. 2014;13:686–699.
24. Chatila ZK, Bradshaw EM. Alzheimer's Disease Genetics: A Dampened Microglial Response? The Neuroscientist. 2021:10738584211024532.
25. Bryan KJ, Zhu X, Harris PL, Perry G, Castellani RJ, Smith MA, et al. Expression of CD74 is increased in neurofibrillary tangles in Alzheimer's disease. Mol Neurodegener. 2008;3:13.

26. Parachikova A, Agadjanyan MG, Cribbs DH, Blurton-Jones M, Perreau V, Rogers J, et al. Inflammatory changes parallel the early stages of Alzheimer disease. Neurobiol Aging. 2007;28:1821–1833.
27. Togo T, Akiyama H, Iseki E, Kondo H, Ikeda K, Kato M, et al. Occurrence of T cells in the brain of Alzheimer's disease and other neurological diseases. J Neuroimmunol. 2002;124:83–92.
28. Merlini M, Kirabali T, Kulic L, Nitsch RM, Ferretti MT. Extravascular CD3+ T Cells in Brains of Alzheimer Disease Patients Correlate with Tau but Not with Amyloid Pathology: An Immunohistochemical Study. Neurodegener Dis. 2018;18:49–56.
29. Khemka VK, Ganguly A, Bagchi D, Ghosh A, Bir A, Biswas A, et al. Raised Serum Proinflammatory Cytokines in Alzheimer's Disease with Depression. Aging Dis. 2014;5:170–176.
30. Postal M, Appenzeller S. The importance of cytokines and autoantibodies in depression. Autoimmun Rev. 2015;14:30–35.
31. Bialek K, Czarny P, Strycharz J, Sliwinski T. Major depressive disorders accompanying autoimmune diseases - Response to treatment. Prog Neuropsychopharmacol Biol Psychiatry. 2019;95:109678.
32. Lawrence T. The Nuclear Factor NF-κB Pathway in Inflammation. Cold Spring Harb Perspect Biol. 2009;1:a001651.
33. Jha NK, Jha SK, Kar R, Nand P, Swati K, Goswami VK. Nuclear factor-kappa β as a therapeutic target for Alzheimer's disease. J Neurochem. 2019;150:113–137.
34. Affram KO, Mitchell K, Symes AJ. Microglial Activation Results in Inhibition of TGF-β-Regulated Gene Expression. J Mol Neurosci MN. 2017;63:308–319.
35. Mir M, Tolosa L, Asensio VJ, Lladó J, Olmos G. Complementary roles of tumor necrosis factor alpha and interferon gamma in inducible microglial nitric oxide generation. J Neuroimmunol. 2008;204:101–109.
36. Oh H, Ghosh S. NF-κB: Roles and Regulation In Different CD4+ T cell subsets. Immunol Rev. 2013;252:41–51.
37. Kovac A, Zilka N, Kazmerova Z, Cente M, Zilkova M, Novak M. Misfolded truncated protein τ induces innate immune response via MAPK pathway. J Immunol Baltim Md 1950. 2011;187:2732–2739.
38. Lyu Q, Wawrzyniuk M, Rutten VPMG, van Eden W, Sijts AJAM, Broere F. Hsp70 and NF-kB Mediated Control of Innate Inflammatory Responses in a Canine Macrophage Cell Line. Int J Mol Sci. 2020;21:6464.
39. Lackie RE, Maciejewski A, Ostapchenko VG, Marques-Lopes J, Choy W-Y, Duennwald ML, et al. The Hsp70/Hsp90 Chaperone Machinery in Neurodegenerative Diseases. Front Neurosci. 2017;11:254.
40. Huang C, Lu X, Wang J, Tong L, Jiang B, Zhang W. Inhibition of endogenous heat shock protein 70 attenuates inducible nitric oxide synthase induction via disruption of heat shock protein 70/Na(+) /H(+) exchanger 1-Ca(2+) -calcium-calmodulin-dependent protein kinase II/transforming growth factor β-activated kinase 1-nuclear factor-κB signals in BV-2 microglia. J Neurosci Res. 2015;93:1192–1202.
41. Nivon M, Fort L, Muller P, Richet E, Simon S, Guey B, et al. NFκB is a central regulator of protein quality control in response to protein aggregation stresses via autophagy modulation. Mol Biol Cell. 2016;27:1712–1727.
42. Bonaiuto C, McDonald PP, Rossi F, Cassatella MA. Activation of nuclear factor-kappa B by beta-amyloid peptides and interferon-gamma in murine microglia. J Neuroimmunol. 1997;77:51–56.
43. Mao Y, Fisher DW, Yang S, Keszycki RM, Dong H. Protein-protein interactions underlying the behavioral and psychological symptoms of dementia (BPSD) and Alzheimer's disease. PLoS ONE. 2020;15.
44. O'Brien WT, Klein PS. Validating GSK3 as an in vivo target of lithium action. Biochem Soc Trans. 2009;37:1133–1138.
45. Harrison PJ, Cipriani A, Harmer CJ, Nobre AC, Saunders K, Goodwin GM, et al. Innovative approaches to bipolar disorder and its treatment. Ann N Y Acad Sci. 2016;1366:76–89.
46. Haussmann R, Noppes F, Brandt MD, Bauer M, Donix M. Minireview: Lithium: a therapeutic option in Alzheimer's disease and its prodromal stages? Neurosci Lett. 2021;760:136044.
47. Tramutola A, Lanzillotta C, Perluigi M, Butterfield DA. Oxidative stress, protein modification and Alzheimer disease. Brain Res Bull. 2017;133:88–96.

48. Sharma C, Kim S, Nam Y, Jung UJ, Kim SR. Mitochondrial Dysfunction as a Driver of Cognitive Impairment in Alzheimer's Disease. Int J Mol Sci. 2021;22:4850.
49. Swerdlow RH. Mitochondria and Mitochondrial Cascades in Alzheimer's Disease. J Alzheimers Dis;62:1403–1416.
50. Ding Q, Markesbery WR, Chen Q, Li F, Keller JN. Ribosome Dysfunction Is an Early Event in Alzheimer's Disease. J Neurosci. 2005;25:9171–9175.
51. Elder MK, Erdjument-Bromage H, Oliveira MM, Mamcarz M, Neubert TA, Klann E. Age-dependent shift in the de novo proteome accompanies pathogenesis in an Alzheimer's disease mouse model. Commun Biol. 2021;4:823.
52. Ding Q, Markesbery WR, Cecarini V, Keller JN. Decreased RNA, and Increased RNA Oxidation, in Ribosomes from Early Alzheimer's Disease. Neurochem Res. 2006;31:705–710.
53. Hernández-Ortega K, Garcia-Esparcia P, Gil L, Lucas JJ, Ferrer I. Altered Machinery of Protein Synthesis in Alzheimer's: From the Nucleolus to the Ribosome. Brain Pathol. 2015;26:593–605.
54. Slomnicki LP, Pietrzak M, Vashishta A, Jones J, Lynch N, Elliot S, et al. Requirement of Neuronal Ribosome Synthesis for Growth and Maintenance of the Dendritic Tree. J Biol Chem. 2016;291:5721–5739.
55. Shigeoka T, Koppers M, Wong HH-W, Lin JQ, Cagnetta R, Dwivedy A, et al. On-Site Ribosome Remodeling by Locally Synthesized Ribosomal Proteins in Axons. Cell Rep. 2019;29:3605-3619.e10.
56. Ma T, Trinh MA, Wexler AJ, Bourbon C, Gatti E, Pierre P, et al. Suppression of eIF2α kinases alleviates AD-related synaptic plasticity and spatial memory deficits. Nat Neurosci. 2013;16:1299–1305.
57. Koren SA, Hamm MJ, Meier SE, Weiss BE, Nation GK, Chishti EA, et al. Tau drives translational selectivity by interacting with ribosomal proteins. Acta Neuropathol (Berl). 2019;137:571–583.
58. Ximerakis M, Lipnick SL, Innes BT, Simmons SK, Adiconis X, Dionne D, et al. Single-cell transcriptomic profiling of the aging mouse brain. Nat Neurosci. 2019;22:1696–1708.
59. Wang X, Chen K, Pan M, Ge W, He Z. Comparison of proteome alterations during aging in the temporal lobe of humans and rhesus macaques. Exp Brain Res. 2020;238:1963–1976.
60. Li Y, Yu H, Chen C, Li S, Zhang Z, Xu H, et al. Proteomic Profile of Mouse Brain Aging Contributions to Mitochondrial Dysfunction, DNA Oxidative Damage, Loss of Neurotrophic Factor, and Synaptic and Ribosomal Proteins. Oxid Med Cell Longev. 2020;2020:5408452.
61. Turi Z, Lacey M, Mistrik M, Moudry P. Impaired ribosome biogenesis: mechanisms and relevance to cancer and aging. Aging. 2019;11:2512–2540.
62. Mortby ME, Maercker A, Forstmeier S. Apathy: a separate syndrome from depression in dementia? A critical review. Aging Clin Exp Res. 2012;24:305–316.
63. Tagariello P, Girardi P, Amore M. Depression and apathy in dementia: same syndrome or different constructs? A critical review. Arch Gerontol Geriatr. 2009;49:246–249.
64. Qin Q, Teng Z, Liu C, Li Q, Yin Y, Tang Y. TREM2, microglia, and Alzheimer's disease. Mech Ageing Dev. 2021;195:111438.
65. Zhao L. CD33 in Alzheimer's Disease - Biology, Pathogenesis, and Therapeutics: A Mini-Review. Gerontology. 2019;65:323–331.
66. Haure-Mirande J-V, Audrain M, Ehrlich ME, Gandy S. Microglial TYROBP/DAP12 in Alzheimer's disease: Transduction of physiological and pathological signals across TREM2. Mol Neurodegener. 2022;17:55.
67. Zhang B, Gaiteri C, Bodea L-G, Wang Z, McElwee J, Podtelezhnikov AA, et al. Integrated Systems Approach Identifies Genetic Nodes and Networks in Late-Onset Alzheimer's Disease. Cell. 2013;153:707–720.
68. Dalakas MC, Alexopoulos H, Spaeth PJ. Complement in neurological disorders and emerging complement-targeted therapeutics. Nat Rev Neurol. 2020;16:601–617.
69. Krance SH, Wu C-Y, Zou Y, Mao H, Toufighi S, He X, et al. The complement cascade in Alzheimer's disease: a systematic review and meta-analysis. Mol Psychiatry. 2021;26:5532–5541.

70. Grossman HT, Sano M, Aloysi A, Elder GA, Neugroschl J, Schimming C, et al. Prevalent, persistent, and impairing: Longitudinal course and impact of apathy in Alzheimer's disease. Alzheimers Dement Diagn Assess Dis Monit. 2022;13:e12169.
71. Landes AM, Sperry SD, Strauss ME. Prevalence of Apathy, Dysphoria, and Depression in Relation to Dementia Severity in Alzheimer's Disease. J Neuropsychiatry Clin Neurosci. 2005;17:342–349.
72. Thal DR, Arendt T, Waldmann G, Holzer M, Zedlick D, Rüb U, et al. Progression of neurofibrillary changes and PHF-τ in end-stage Alzheimer's disease is different from plaque and cortical microglial pathology. Neurobiol Aging. 1998;19:517–525.
73. Mrak RE. Microglia in Alzheimer brain: a neuropathological perspective. Int J Alzheimers Dis. 2012;2012:165021.
74. Romero-Molina C, Garretti F, Andrews SJ, Marcora E, Goate AM. Microglial efferocytosis: Diving into the Alzheimer's disease gene pool. Neuron. 2022;110:3513–3533.
75. Lamothe B, Besse A, Campos AD, Webster WK, Wu H, Darnay BG. Site-specific Lys-63-linked Tumor Necrosis Factor Receptor-associated Factor 6 Auto-ubiquitination Is a Critical Determinant of IκB Kinase Activation*. J Biol Chem. 2007;282:4102–4112.
76. Sarikas A, Hartmann T, Pan Z-Q. The cullin protein family. Genome Biol. 2011;12:220.
77. Watanabe Y, Taguchi K, Tanaka M. Ubiquitin, Autophagy and Neurodegenerative Diseases. Cells. 2020;9:2022.
78. Bhutani S, Das A, Maheshwari M, Lakhotia SC, Jana NR. Dysregulation of core components of SCF complex in poly-glutamine disorders. Cell Death Dis. 2012;3:e428.
79. Zucchelli S, Marcuzzi F, Codrich M, Agostoni E, Vilotti S, Biagioli M, et al. Tumor Necrosis Factor Receptor-associated Factor 6 (TRAF6) Associates with Huntingtin Protein and Promotes Its Atypical Ubiquitination to Enhance Aggregate Formation*. J Biol Chem. 2011;286:25108–25117.
80. Chen ZS, Wong AKY, Cheng TC, Koon AC, Chan HYE. FipoQ/FBXO33, a Cullin-1-based ubiquitin ligase complex component modulates ubiquitination and solubility of polyglutamine disease protein. J Neurochem. 2019;149:781–798.
81. Gerez JA, Prymaczok NC, Rockenstein E, Herrmann US, Schwarz P, Adame A, et al. A cullin-RING ubiquitin ligase targets exogenous α-synuclein and inhibits Lewy body-like pathology. Sci Transl Med. 2019;11:eaau6722.
82. Zucchelli S, Codrich M, Marcuzzi F, Pinto M, Vilotti S, Biagioli M, et al. TRAF6 promotes atypical ubiquitination of mutant DJ-1 and alpha-synuclein and is localized to Lewy bodies in sporadic Parkinson's disease brains. Hum Mol Genet. 2010;19:3759–3770.
83. Liu D, Dai S-X, He K, Li G-H, Liu J, Liu LG, et al. Identification of hub ubiquitin ligase genes affecting Alzheimer's disease by analyzing transcriptome data from multiple brain regions. Sci Prog. 2021;104:00368504211001146.
84. Chen Y, Neve RL, Liu H. Neddylation dysfunction in Alzheimer's disease. J Cell Mol Med. 2012;16:2583–2591.
85. Potjewyd FM, Axtman AD. Exploration of Aberrant E3 Ligases Implicated in Alzheimer's Disease and Development of Chemical Tools to Modulate Their Function. Front Cell Neurosci. 2021;15:768655.
86. El Idrissi F, Gressier B, Devos D, Belarbi K. A Computational Exploration of the Molecular Network Associated to Neuroinflammation in Alzheimer's Disease. Front Pharmacol. 2021;12:630003.
87. Wang S, Zhang X, Zhai L, Sheng X, Zheng W, Chu H, et al. Atorvastatin Attenuates Cognitive Deficits and Neuroinflammation Induced by Aβ1–42 Involving Modulation of TLR4/TRAF6/NF-κB Pathway. J Mol Neurosci. 2018;64:363–373.
88. Yan R, Farrelly S, McCarthy JV. Presenilins are novel substrates for TRAF6-mediated ubiquitination. Cell Signal. 2013;25:1769–1779.
89. Babu JR, Geetha T, Wooten MW. Sequestosome 1/p62 shuttles polyubiquitinated tau for proteasomal degradation. J Neurochem. 2005;94:192–203.
90. Geetha T, Zheng C, McGregor WC, Douglas White B, Diaz-Meco MT, Moscat J, et al. TRAF6 and p62 inhibit amyloid β-induced neuronal death through p75 neurotrophin receptor. Neurochem Int. 2012;61:1289–1293.

91. Kato A, Rouach N, Nicoll RA, Bredt DS. Activity-dependent NMDA receptor degradation mediated by retrotranslocation and ubiquitination. Proc Natl Acad Sci. 2005;102:5600–5605.
92. Kumar D, Ambasta RK, Kumar P. Ubiquitin biology in neurodegenerative disorders: From impairment to therapeutic strategies. Ageing Res Rev. 2020;61:101078.
93. Dancy BM, Cole PA. Protein Lysine Acetylation by p300/CBP. Chem Rev. 2015;115:2419–2452.
94. Schueller E, Paiva I, Blanc F, Wang X-L, Cassel J-C, Boutillier A-L, et al. Dysregulation of histone acetylation pathways in hippocampus and frontal cortex of Alzheimer’s disease patients. Eur Neuropsychopharmacol. 2020;33:101–116.
95. Song H, Moon M, Choe HK, Han D-H, Jang C, Kim A, et al. Aβ-induced degradation of BMAL1 and CBP leads to circadian rhythm disruption in Alzheimer’s disease. Mol Neurodegener. 2015;10:13.
96. Caccamo A, Maldonado MA, Bokov AF, Majumder S, Oddo S. CBP gene transfer increases BDNF levels and ameliorates learning and memory deficits in a mouse model of Alzheimer’s disease. Proc Natl Acad Sci U S A. 2010;107:22687–22692.
97. Chatterjee S, Cassel R, Schneider-Anthony A, Merienne K, Cosquer B, Tzeplaeff L, et al. Reinstating plasticity and memory in a tauopathy mouse model with an acetyltransferase activator. EMBO Mol Med. 2018;10:e8587.
98. Lu X, Deng Y, Yu D, Cao H, Wang L, Liu L, et al. Histone acetyltransferase p300 mediates histone acetylation of PS1 and BACE1 in a cellular model of Alzheimer’s disease. PloS One. 2014;9:e103067.
99. Chen X, Li Y, Wang C, Tang Y, Mok S-A, Tsai RM, et al. Promoting tau secretion and propagation by hyperactive p300/CBP via autophagy-lysosomal pathway in tauopathy. Mol Neurodegener. 2020;15:2.
100. Aubry S, Shin W, Crary JF, Lefort R, Qureshi YH, Lefebvre C, et al. Assembly and Interrogation of Alzheimer’s Disease Genetic Networks Reveal Novel Regulators of Progression. PLoS ONE. 2015;10:e0120352.
101. Sen T, Saha P, Sen N. Nitrosylation of GAPDH augments pathological tau acetylation upon exposure to amyloid-β. Sci Signal. 2018;11:eaao6765.
102. Min S-W, Cho S-H, Zhou Y, Schroeder S, Haroutunian V, Seeley WW, et al. Acetylation of tau inhibits its degradation and contributes to tauopathy. Neuron. 2010;67:953–966.
103. Portillo M, Eremenko E, Kaluski S, Garcia-Venzor A, Onn L, Stein D, et al. SIRT6-CBP-dependent nuclear Tau accumulation and its role in protein synthesis. Cell Rep. 2021;35:109035.
104. Shin M-K, Vázquez-Rosa E, Koh Y, Dhar M, Chaubey K, Cintrón-Pérez CJ, et al. Reducing acetylated-tau is neuroprotective in brain injury. Cell. 2021;184:2715-2732.e23.
105. Worthington A, Wood RL. Apathy following traumatic brain injury: A review. Neuropsychologia. 2018;118:40–47.
106. Dickey CA, Dunmore J, Lu B, Wang J-W, Lee WC, Kamal A, et al. HSP induction mediates selective clearance of tau phosphorylated at proline-directed Ser/Thr sites but not KXGS (MARK) sites. FASEB J Off Publ Fed Am Soc Exp Biol. 2006;20:753–755.
107. Blair LJ, Nordhues BA, Hill SE, Scaglione KM, O’Leary JC, Fontaine SN, et al. Accelerated neurodegeneration through chaperone-mediated oligomerization of tau. J Clin Invest. 2013;123:4158–4169.
108. Klein H-U, McCabe C, Gjoneska E, Sullivan SE, Kaskow BJ, Tang A, et al. Epigenome-wide study uncovers large-scale changes in histone acetylation driven by tau pathology in aging and Alzheimer’s human brains. Nat Neurosci. 2019;22:37–46.
109. Beaulieu J-M, Gainetdinov RR. The Physiology, Signaling, and Pharmacology of Dopamine Receptors. Pharmacol Rev. 2011;63:182–217.
110. Thibault D, Albert PR, Pineyro G, Trudeau L-É. Neurotensin triggers dopamine D2 receptor desensitization through a protein kinase C and beta-arrestin1-dependent mechanism. J Biol Chem. 2011;286:9174–9184.
111. Zhang X, Min X, Zhu A, Kim K-M. A novel molecular mechanism involved in the crosstalks between homologous and PKC-mediated heterologous regulatory pathway of dopamine D2 receptor. Biochem Pharmacol. 2020;174:113791.

112. Qureshi HY, Li T, MacDonald R, Cho CM, Leclerc N, Paudel HK. Interaction of 14-3-3ζ with microtubule-associated protein tau within Alzheimer's disease neurofibrillary tangles. Biochemistry. 2013;52:6445–6455.
113. Canet G, Chevallier N, Zussy C, Desrumaux C, Givalois L. Central Role of Glucocorticoid Receptors in Alzheimer's Disease and Depression. Front Neurosci. 2018;12:739.
114. Koyuncu S, Fatima A, Gutierrez-Garcia R, Vilchez D. Proteostasis of Huntingtin in Health and Disease. Int J Mol Sci. 2017;18:1568.
115. McKeith IG, Boeve BF, Dickson DW, Halliday G, Taylor J-P, Weintraub D, et al. Diagnosis and management of dementia with Lewy bodies: Fourth consensus report of the DLB Consortium. Neurology. 2017;89:88–100.
116. Rossi M, Farcy N, Starkstein SE, Merello M. Nosology and Phenomenology of Psychosis in Movement Disorders. Mov Disord Clin Pract. 2020;7:140–153.
117. Schoretsanitis G, de Filippis R, Ntogka M, Leucht S, Correll CU, Kane JM. Matrix Metalloproteinase 9 Blood Alterations in Patients With Schizophrenia Spectrum Disorders: A Systematic Review and Meta-Analysis. Schizophr Bull. 2021;47:986–996.
118. Vizlin-Hodzic D, Zhai Q, Illes S, Södersten K, Truvé K, Parris TZ, et al. Early onset of inflammation during ontogeny of bipolar disorder: the NLRP2 inflammasome gene distinctly differentiates between patients and healthy controls in the transition between iPS cell and neural stem cell stages. Transl Psychiatry. 2017;7:e1010.
119. Alkelai A, Lupoli S, Greenbaum L, Kohn Y, Kanyas-Sarner K, Ben-Asher E, et al. DOCK4 and CEACAM21 as novel schizophrenia candidate genes in the Jewish population. Int J Neuropsychopharmacol. 2012;15:459–469.
120. Edwards AC, Bacanu S-A, Bigdeli TB, Moscati A, Kendler KS. Evaluating the dopamine hypothesis of schizophrenia in a large-scale genome-wide association study. Schizophr Res. 2016;176:136–140.
121. Mühlbauer V, Möhler R, Dichter MN, Zuidema SU, Köpke S, Luijendijk HJ. Antipsychotics for agitation and psychosis in people with Alzheimer's disease and vascular dementia. Cochrane Database Syst Rev. 2021;12:CD013304.
122. Stockmeier CA, DiCarlo JJ, Zhang Y, Thompson P, Meltzer HY. Characterization of typical and atypical antipsychotic drugs based on in vivo occupancy of serotonin2 and dopamine2 receptors. J Pharmacol Exp Ther. 1993;266:1374–1384.
123. Montalvo-Ortiz JL, Fisher DW, Rodríguez G, Fang D, Csernansky JG, Dong H. Histone deacetylase inhibitors reverse age-related increases in side effects of haloperidol in mice. Psychopharmacology (Berl). 2017;234:2385–2398.
124. Montalvo-Ortiz JL, Keegan J, Gallardo C, Gerst N, Tetsuka K, Tucker C, et al. HDAC Inhibitors Restore the Capacity of Aged Mice to Respond to Haloperidol through Modulation of Histone Acetylation. Neuropsychopharmacology. 2014;39:1469–1478.
125. Koenig AM, Arnold SE, Streim JE. Agitation and Irritability in Alzheimer's Disease: Evidenced-Based Treatments and the Black-Box Warning. Curr Psychiatry Rep. 2016;18:3.
126. Jacoby E, Bouhelal R, Gerspacher M, Seuwen K. The 7 TM G-Protein-Coupled Receptor Target Family. ChemMedChem. 2006;1:760–782.
127. Beaulieu J-M, Espinoza S, Gainetdinov RR. Dopamine receptors – IUPHAR Review 13. Br J Pharmacol. 2015;172:1–23.
128. van Gastel J, Leysen H, Boddaert J, vangenechten L, Luttrell LM, Martin B, et al. Aging-related modifications to G protein-coupled receptor signaling diversity. Pharmacol Ther. 2021;223:107793.
129. Jiang T, Yu J-T, Tan M-S, Zhu X-C, Tan L. β-Arrestins as Potential Therapeutic Targets for Alzheimer's Disease. Mol Neurobiol. 2013;48:812–818.
130. Woo J-AA, Liu T, Fang CC, Castaño MA, Kee T, Yrigoin K, et al. β-Arrestin2 oligomers impair the clearance of pathological tau and increase tau aggregates. Proc Natl Acad Sci U S A. 2020;117:5006–5015.

131. Woo J-A, Yan Y, Kee TR, Cazzaro S, McGill Percy KC, Wang X, et al. β-arrestin1 promotes tauopathy by transducing GPCR signaling, disrupting microtubules and autophagy. Life Sci Alliance. 2022;5:e202101183.
132. Oakley RH, Laporte SA, Holt JA, Caron MG, Barak LS. Differential affinities of visual arrestin, beta arrestin1, and beta arrestin2 for G protein-coupled receptors delineate two major classes of receptors. J Biol Chem. 2000;275:17201–17210.
133. Pair FS, Yacoubian TA. 14-3-3 Proteins: Novel Pharmacological Targets in Neurodegenerative Diseases. Trends Pharmacol Sci. 2021;42:226–238.
134. Cau Y, Valensin D, Mori M, Draghi S, Botta M. Structure, Function, Involvement in Diseases and Targeting of 14-3-3 Proteins: An Update. Curr Med Chem. 2018;25:5–21.
135. Shimada T, Fournier AE, Yamagata K. Neuroprotective function of 14-3-3 proteins in neurodegeneration. BioMed Res Int. 2013;2013:564534.
136. Jia B, Wu Y, Zhou Y. 14-3-3 and aggresome formation: implications in neurodegenerative diseases. Prion. 2014;8:28123.
137. Yuan L, Barbash S, Kongsamut S, Eishingdrelo A, Sakmar TP, Eishingdrelo H. 14-3-3 signal adaptor and scaffold proteins mediate GPCR trafficking. Sci Rep. 2019;9:11156.
138. Eishingdrelo H, Qin X, Yuan L, Kongsamut S, Yu L. Ligands can differentially and temporally modulate GPCR interaction with 14-3-3 isoforms. Curr Res Pharmacol Drug Discov. 2022;3:100123.
139. McFerrin MB, Chi X, Cutter G, Yacoubian TA. Dysregulation of 14-3-3 proteins in neurodegenerative diseases with Lewy body or Alzheimer pathology. Ann Clin Transl Neurol. 2017;4:466–477.
140. Umahara T, Uchihara T, Tsuchiya K, Nakamura A, Iwamoto T, Ikeda K, et al. 14-3-3 proteins and zeta isoform containing neurofibrillary tangles in patients with Alzheimer's disease. Acta Neuropathol (Berl). 2004;108:279–286.
141. Hashiguchi M, Sobue K, Paudel HK. 14-3-3zeta is an effector of tau protein phosphorylation. J Biol Chem. 2000;275:25247–25254.
142. Ramshaw H, Xu X, Jaehne EJ, McCarthy P, Greenberg Z, Saleh E, et al. Locomotor hyperactivity in 14-3-3ζ KO mice is associated with dopamine transporter dysfunction. Transl Psychiatry. 2013;3:e327–e327.
143. Zuehlke AD, Beebe K, Neckers L, Prince T. Regulation and function of the human HSP90AA1 gene. Gene. 2015;570:8–16.
144. Murray PS, Kumar S, DeMichele-Sweet MAA, Sweet RA. Psychosis in Alzheimer's Disease. Biol Psychiatry. 2014;75:542–552.
145. Gomar JJ, Tan G, Halpern J, Gordon ML, Greenwald B, Koppel J. Increased retention of tau PET ligand [18F]-AV1451 in Alzheimer's Disease Psychosis. Transl Psychiatry. 2022;12:82.
146. O'Rourke L, Murphy KC. Recent developments in understanding the relationship between 22q11.2 deletion syndrome and psychosis. Curr Opin Psychiatry. 2019;32:67–72.
147. Namavar Y, Duineveld DJ, Both GIA, Fiksinski AM, Vorstman JAS, Verhoeven-Duif NM, et al. Psychiatric phenotypes associated with hyperprolinemia: A systematic review. Am J Med Genet B Neuropsychiatr Genet. 2021;186:289–317.
148. Wang G, Zhou Y, Huang F-J, Tang H-D, Xu X-H, Liu J-J, et al. Plasma Metabolite Profiles of Alzheimer's Disease and Mild Cognitive Impairment. J Proteome Res. 2014;13:2649–2658.
149. Chatterjee P, Cheong Y-J, Bhatnagar A, Goozee K, Wu Y, McKay M, et al. Plasma metabolites associated with biomarker evidence of neurodegeneration in cognitively normal older adults. J Neurochem. 2021;159:389–402.
150. Crabtree GW, Park AJ, Gordon JA, Gogos JA. Cytosolic Accumulation of L-Proline Disrupts GABA-Ergic Transmission through GAD Blockade. Cell Rep. 2016;17:570–582.
151. Raudvere U, Kolberg L, Kuzmin I, Arak T, Adler P, Peterson H, et al. g:Profiler: a web server for functional enrichment analysis and conversions of gene lists (2019 update). Nucleic Acids Res. 2019;47:W191–W198.
152. Zhang B, Horvath S. A general framework for weighted gene co-expression network analysis. Stat Appl Genet Mol Biol. 2005;4:Article17.

153. Langfelder P, Horvath S. WGCNA: an R package for weighted correlation network analysis. BMC Bioinformatics. 2008;9:559.
154. Abbassi-Daloii T, Kan HE, Raz V, 't Hoen PAC. Recommendations for the analysis of gene expression data to identify intrinsic differences between similar tissues. Genomics. 2020;112:3157–3165.
155. da Rocha EL, Ung CY, McGehee CD, Correia C, Li H. NetDecoder: a network biology platform that decodes context-specific biological networks and gene activities. Nucleic Acids Res. 2016;44:e100.
