# Supplementary material for: Unique Transcriptional Signatures Correlate with Behavioral and Psychological Symptom Domains in Alzheimer’s Disease
Source: Res Sq. 2023 Jan 11:rs.3.rs-2444391. Preprint. [Version 1] doi: 10.21203/rs.3.rs-2444391/v1 (PMC9882691; doi:10.21203/rs.3.rs-2444391/v1)
Supplement: 6 — Supplementary Fig.S1 BPSD can be split into four domains based on clustering of symptoms. A) Principal component analysis was performed on the symptom questions from the structured interview for each participant, and a Scree plot was constructed to evaluate how many factors optimally grouped BPSD. The curve began to asymptote between 3-5 factors. B) Horn’s parallel analysis suggested a 4-factor solution, where eigenvalues > 1. C) Each BPSD question was loaded onto a predominant factor, and these loadings were used to sort each question into BPSD domains. D,E) Variance explained by each factor and correlations between factors. Supplementary Fig. S2 Estimated cell abundance for affective, apathy, and psychosis domains. A) Relative cell abundance estimated with BRETIGIA for affective, B) apathy, C) and psychosis domains. After correcting for multiple comparisons, there was a significant decrease in microglia detected in Apathy domain cases (p < 0.05) but not for any other comparison. Supplementary Fig. S3 Agitation domain case subnetwork. Main differences in case subnetwork compared to control subnetwork. Source genes are indicated as diamonds, intermediate genes as circles, and target genes as squares. The color of the node depicts flow difference, with red being high flow in case but low in control and blue being high flow in control but low in case. Edge colors correspond to directionality of correlation, with red being positive and blue being negative. To aid visualization, nodes and edges with highest differences in information flow are shown. Supplementary Fig. S4 Divergent information flow between transcriptional networks in the affective domain. A) Total edge flow profiles in control versus case subnetworks for the affective domain. Overall, edges display decreased flow in cases versus controls. B) Jaccard index evaluating similarity between case and control subnetworks. C) Venn diagrams depicting overlap (blue) in genes, edges, and paths between cases (yellow) [file NIHPPrs2444391v1-supplement-6.pdf]

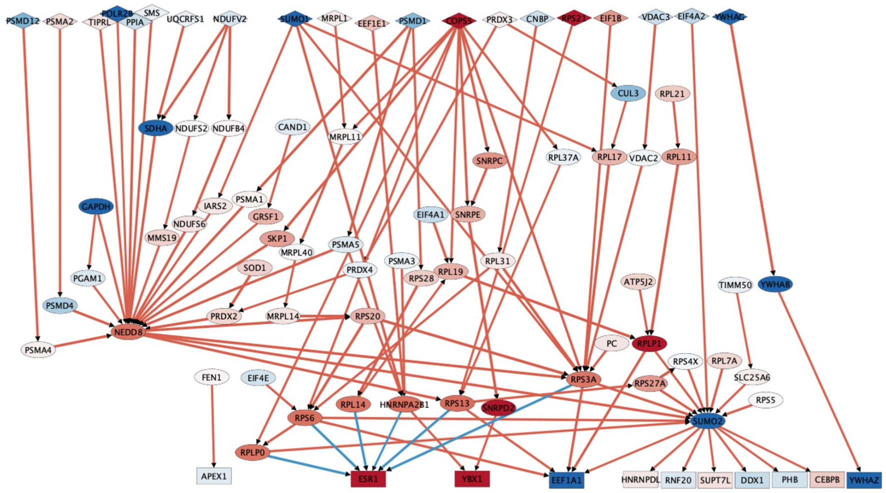

S Fig3

Supplementary Figure 3

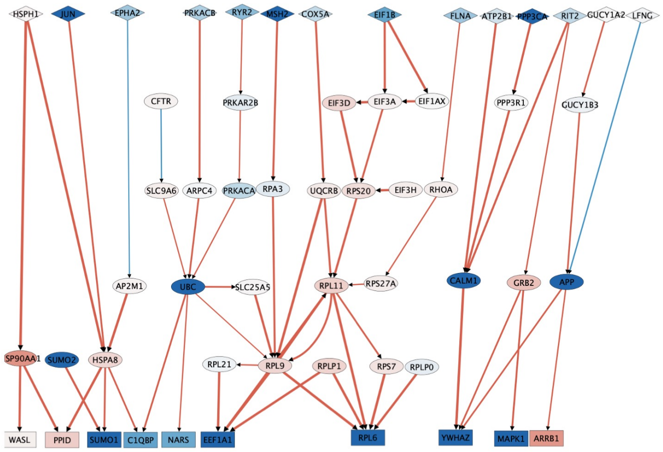

S Fig 10

Supplementary Figure 10

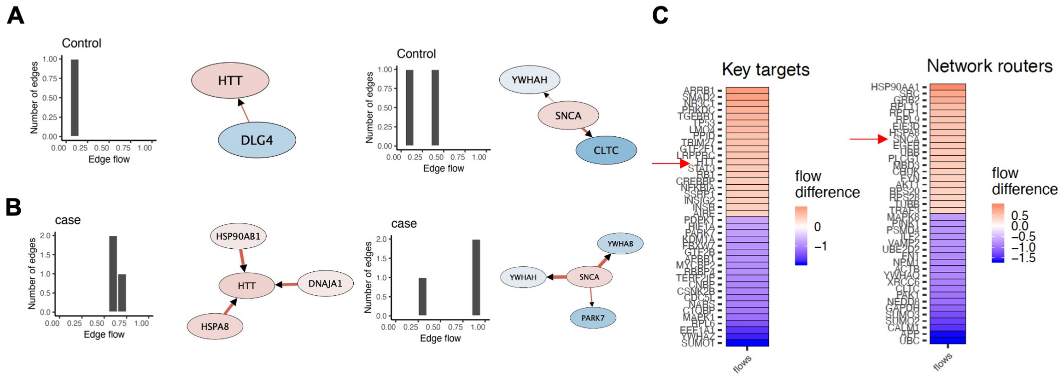

S Fig 9

Supplementary Figure 9

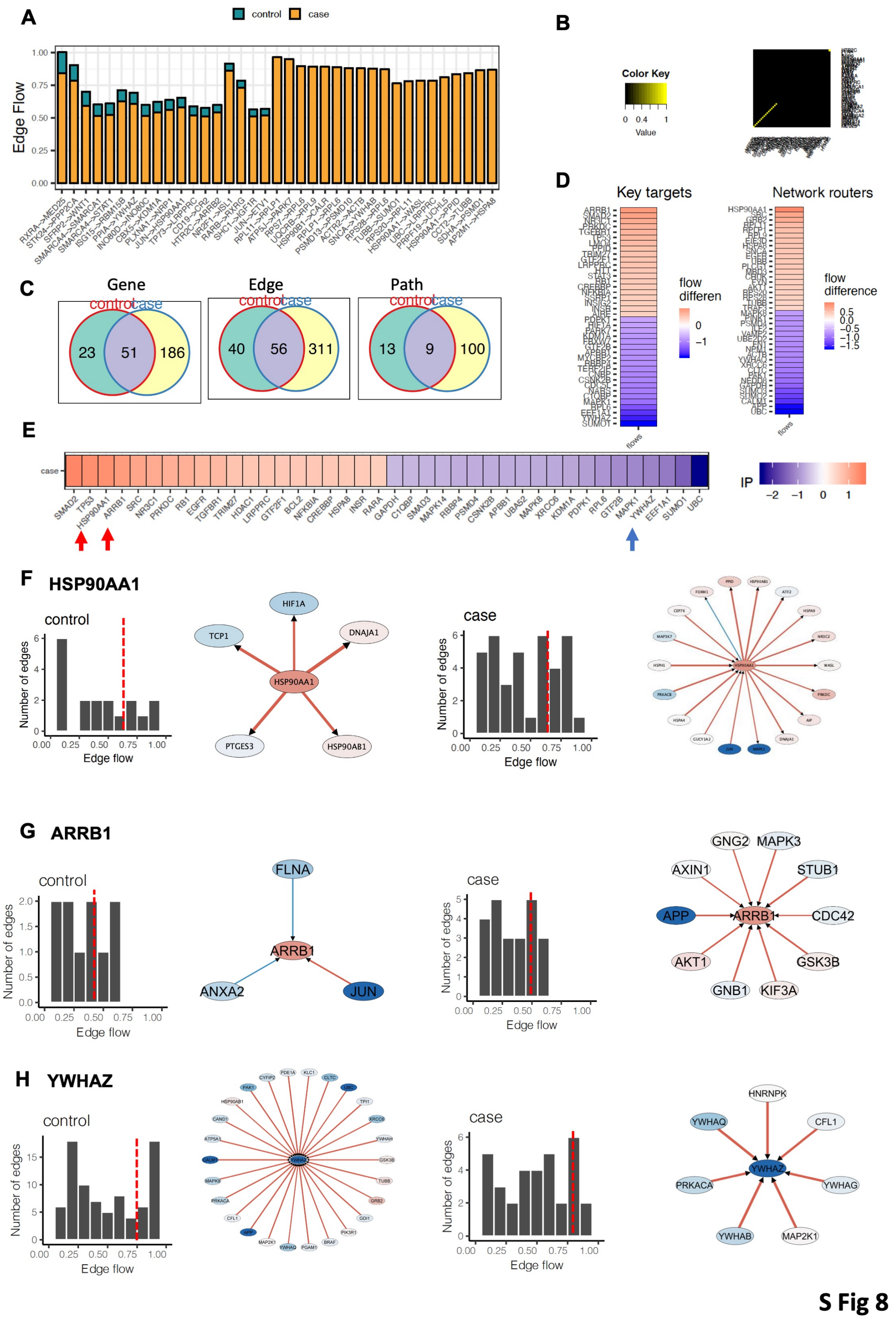

Supplementary Figure 8

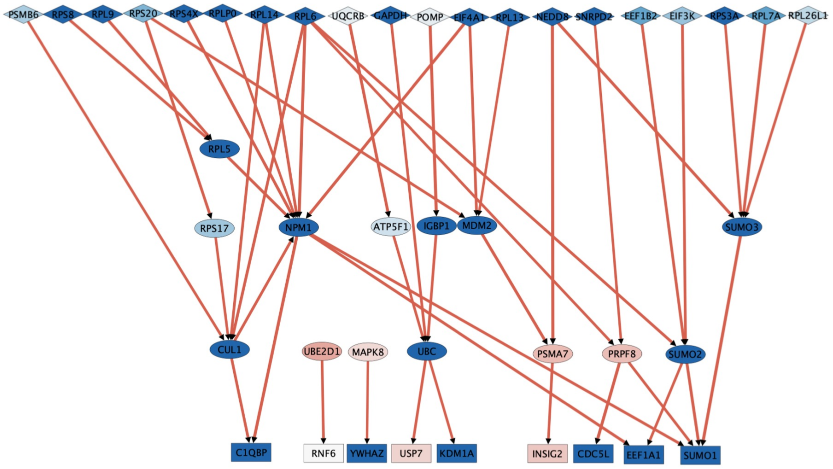

S Fig 7

Supplementary Figure 7

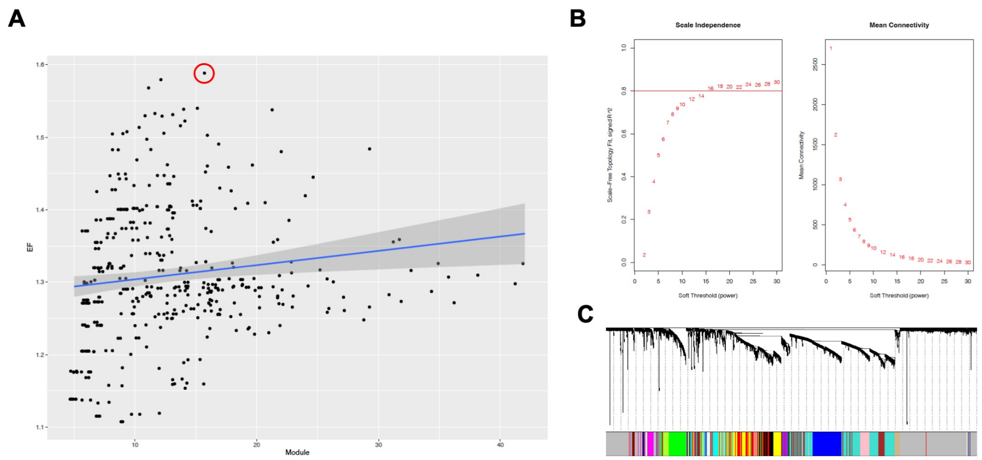

S Fig 11

Supplementary Figure 11

A

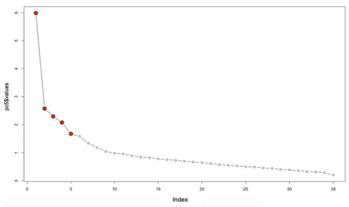

B

```
Results of Horn's Parallel Analysis for component retention
1050 iterations, using the 95 centile estimate

--------------------------------------------------
Component   Adjusted    Unadjusted    Estimated
            Eigenvalue  Eigenvalue    Bias
--------------------------------------------------
1           3.202146    6.159244      2.957097
2           2.563985    4.984237      2.420251
3           1.796578    3.855475      2.058896
4           1.060288    2.846568      1.786280
--------------------------------------------------

Adjusted eigenvalues > 1 indicate dimensions to retain.
(4 components retained)
```

C

	item	TC4	TC1	TC2	TC3	h2	u2	com
halluci10	34	0.71				0.51	0.49	1.0
halluci8	33	0.70				0.50	0.50	1.0
halluci12	35	0.70				0.48	0.52	1.1
halluci6	32	0.66				0.49	0.51	1.3
halluci2	30	0.52				0.36	0.64	1.5
halluci4	31	0.48				0.25	0.75	1.1
fidget4	11	0.45				0.27	0.73	1.4
fidget8	12	0.38				0.27	0.73	1.8
fidget2	10	0.37				0.24	0.76	1.6
delusi4	27					0.12	0.88	1.7
temper9	23		0.79			0.62	0.38	1.0
temper13	25		0.73			0.53	0.47	1.1
temper11	24		0.73			0.50	0.50	1.1
temper4	20		0.66			0.47	0.53	1.1
temper2	19		0.60			0.49	0.51	1.4
temper6	21		0.53			0.32	0.68	1.1
temper8	22		0.45			0.32	0.68	1.5
depress	1			0.75		0.59	0.41	1.1
mood	2			0.69		0.52	0.48	1.2
sadlong	3			0.67		0.48	0.52	1.1
upsetdur	7		0.32	0.47		0.42	0.58	1.9
critical	4			0.46		0.22	0.78	1.2
talkphys	9			0.46		0.22	0.78	1.1
notworth	5			0.43		0.19	0.81	1.2
upset	6			0.41		0.32	0.68	2.1
delusi2	26	0.33		0.37		0.29	0.71	2.7
physanx	8			0.37		0.18	0.82	1.5
delusi8	29					0.14	0.86	2.4
delusi6	28					0.15	0.85	2.2
apathy6	15				0.78	0.64	0.36	1.0
apathy5	14				0.70	0.55	0.45	1.1
apathy10.11	17				0.64	0.46	0.54	1.1
apathy7	16				0.56	0.41	0.59	1.3
apathy12	18				0.50	0.26	0.74	1.1
apathy1	13				0.36	0.15	0.85	1.6

D

	TC4	TC1	TC2	TC3
SS loadings	3.56	3.47	3.28	2.61
Proportion Var	0.10	0.10	0.09	0.07
Cumulative Var	0.10	0.20	0.29	0.37
Proportion Explained	0.28	0.27	0.25	0.20
Cumulative Proportion	0.28	0.54	0.80	1.00

E

	TC4	TC1	TC2	TC3
TC4	1.00	0.28	0.14	0.17
TC1	0.28	1.00	0.17	0.16
TC2	0.14	0.17	1.00	0.18
TC3	0.17	0.16	0.18	1.00

TC1 : Aggression domain
TC2: Affective domain
TC3: Apathy domain
TC4: Psychosis domain

S Fig1

Supplementary Figure 1

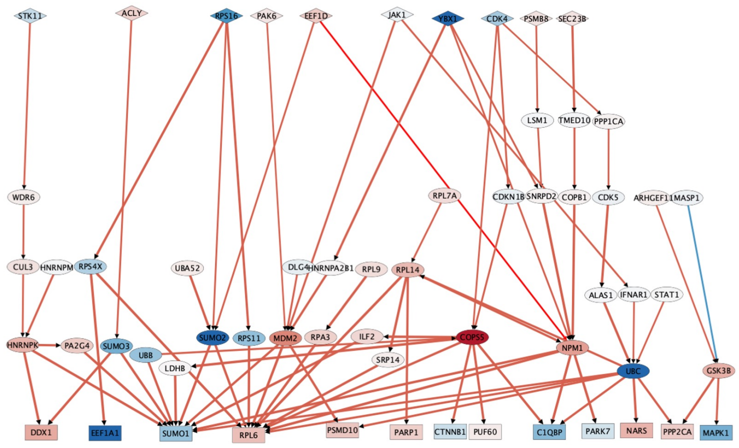

S Fig 5

Supplementary Figure 5

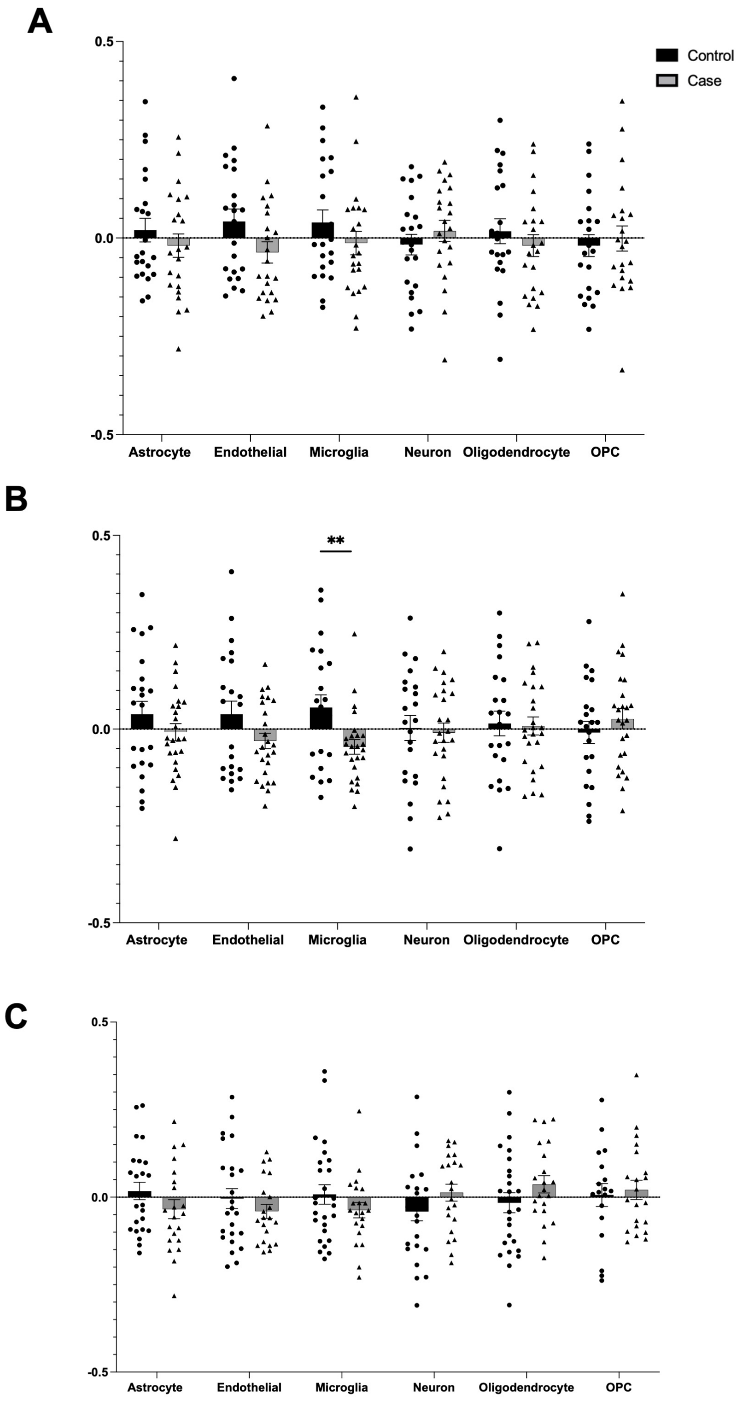

S Fig2

Supplementary Figure 2

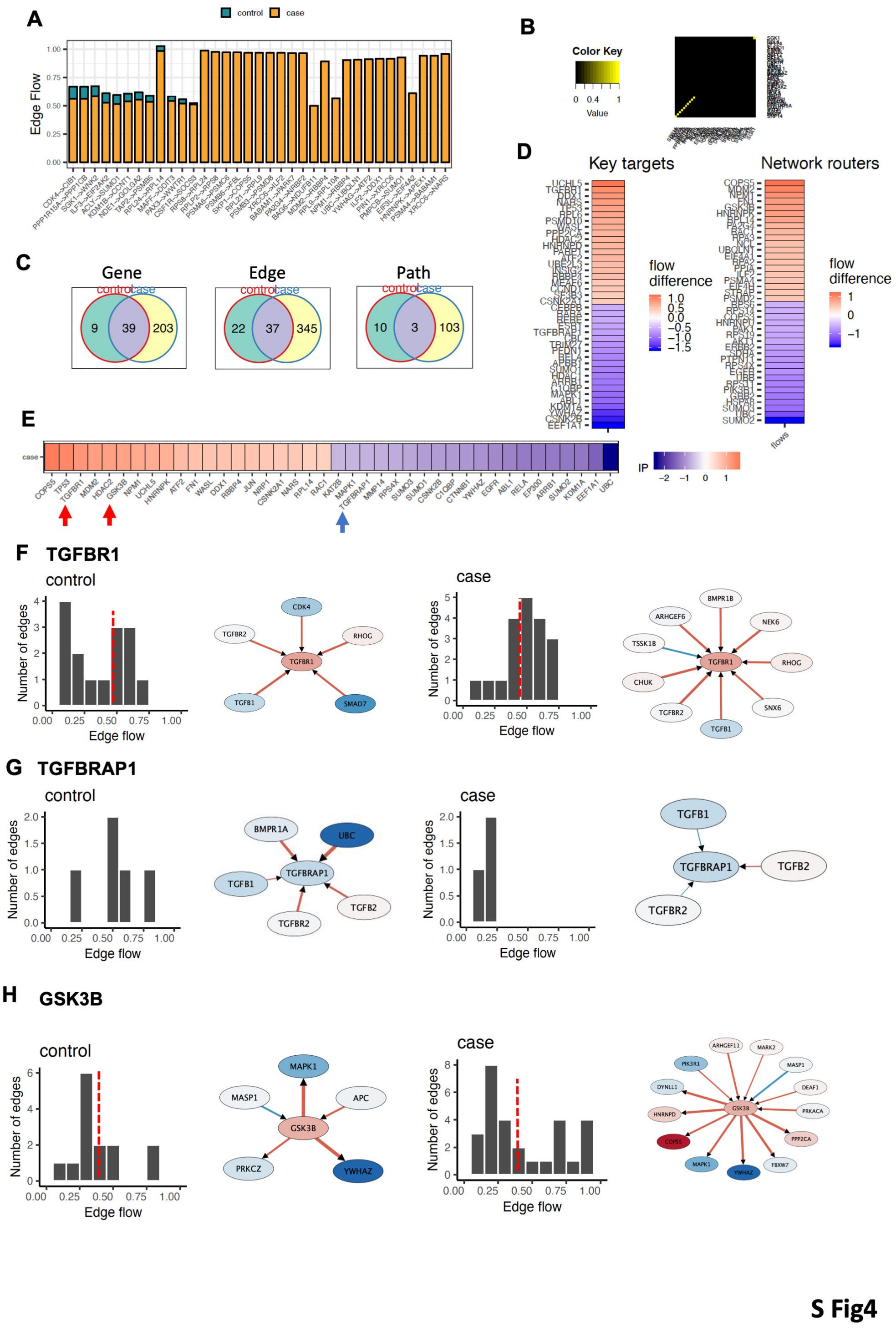

Supplementary Figure 4

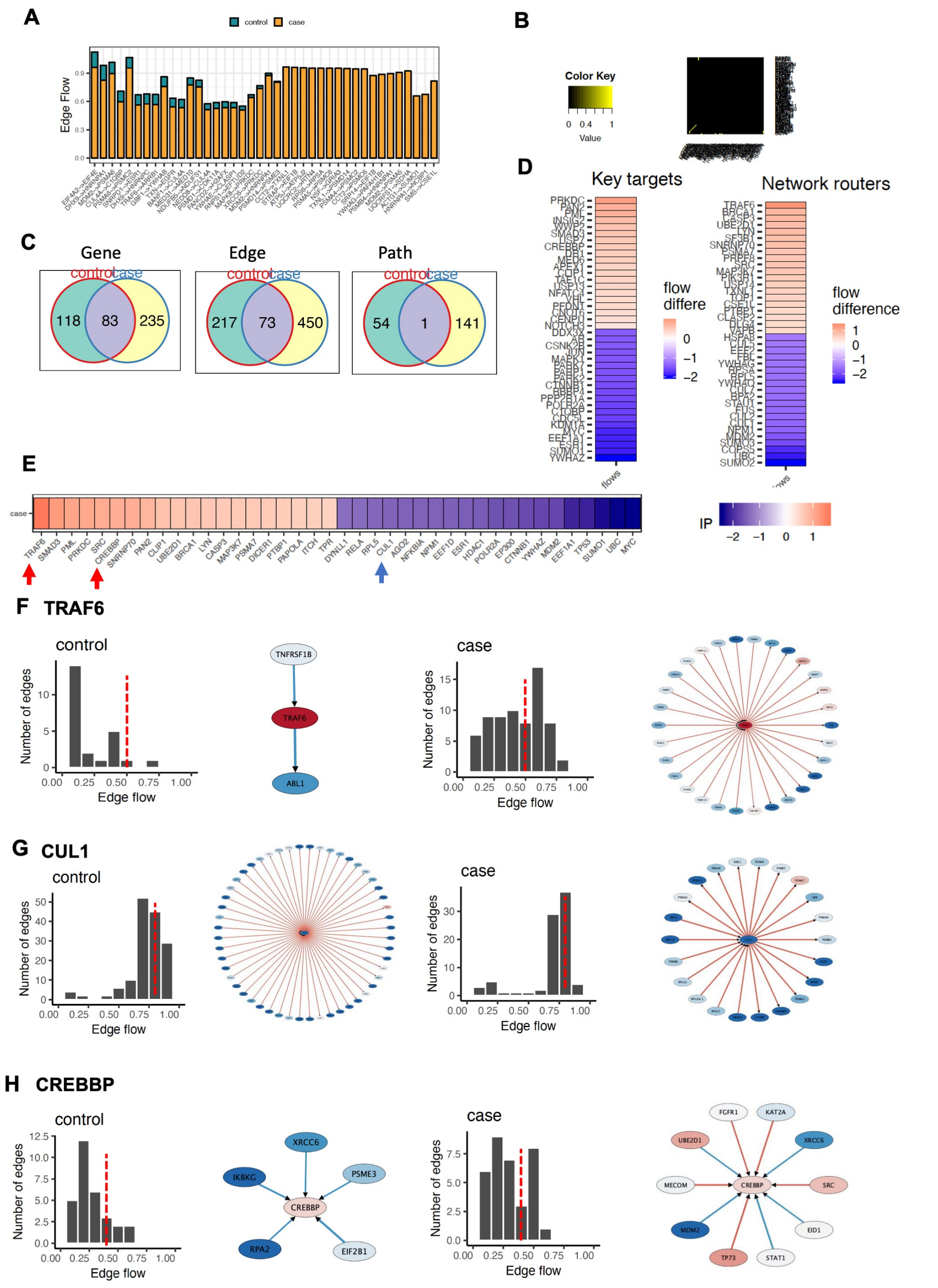

S Fig 6

Supplementary Figure 6
